# Psychological and behavioral profiles of combustible vs. e-cigarette users: a narrative review of the implications for tailored non-pharmacological cessation interventions

**DOI:** 10.3389/fpubh.2026.1779824

**Published:** 2026-06-17

**Authors:** Tae Rim Kim, In Jin Seo, Jeong Soo Kim, Eun-Seung Yu, Hye Ri Kim, Leah Han, Sun Mi Kim

**Affiliations:** 1 Department of Psychiatry, College of Medicine, Chung-Ang University, Seoul, Republic of Korea; 2 School of Individualized Study, Rochester Institute of Technology, Rochester, NY, United States; 3 Department of Counseling Psychology, The Cyber University of Korea, Seoul, Republic of Korea; 4 Boston College, Chestnut Hill, MA, United States

**Keywords:** cognitive behavioral therapy, digital health, e-cigarette, motivational interviewing, smoking cessation, vaping

## Abstract

**Background and aim:**

The decline in combustible cigarette use along with the rapid rise in e-cigarette use necessitates differentiated cessation strategies for each. This narrative review compares psychological, behavioral, and intervention-related characteristics between combustible cigarette users (CCUs) and e-cigarette users (ECUs).

**Methods:**

A structured literature search (2010–2025) of PubMed, Scopus, Embase, and Google Scholar identified 76 representative studies that examined the psychological, behavioral, and intervention-related characteristics of CCUs and ECUs.

**Results:**

CCUs and ECUs may demonstrate distinct dependence patterns and motivational profiles. CCUs tend to exhibit physiological withdrawal and conditioned automaticity, responding best to structured, high-intensity interventions that combine cognitive behavioral therapy with pharmacotherapy. ECUs appear to show reinforcement and lower risk perception, demonstrating greater engagement with digital interventions featuring gamification, peer components, and flexible goal-setting.

**Discussion:**

Effective tobacco control requires a user-specific strategy. For CCUs, multicomponent approaches that emphasize complete cessation, relapse prevention, and withdrawal management are essential. Digital platforms with stepwise goals, peer-based strategies, and cognitive reframing of perceived control are more effective for ECUs. Age-tailored approaches and their systematic integration into healthcare systems are critical for implementation. The findings identify priorities for future intervention research and implementation, and inform a differentiated behavioral framework for tailoring non-pharmacological cessation strategies to CCUs and ECUs.

## 1 Introduction

Smoking is a major global public health concern, causing over 7 million deaths annually (1). It contributes substantially to mortality through its association with cardiovascular and respiratory diseases, including chronic obstructive pulmonary disease, asthma, interstitial lung disease, ischemic heart disease, and heart failure (2). According to the Global Burden of Disease Study 2019, smoking causes approximately 8.71 million deaths and 230 million disability-adjusted life years, with 36.7% of smoking-related deaths being attributed to cardiovascular conditions (3). These health impacts also impose major economic costs, resulting in over one trillion U.S. dollars in global economic losses annually, approximately 40% of which occur in low- and middle-income countries (4).

Nevertheless, smoking has generally reduced over the last few decades. The global adult prevalence of smoking tobacco products (e.g., combustible cigarettes, pipes, cigars, and waterpipes) has declined from 34.3% in 2000 to 22.6% in 2020 [i.e., 11.7% reduction; GBD 2019 Tobacco Collaborators (5)]. High-income countries saw reductions exceeding 40%, with some, such as Brazil, reporting declines of over 70% (5). By contrast, e-cigarette use has increased across all age groups, particularly among adolescents and young adults. In fact, estimates show that more than 100 million people worldwide now use e-cigarettes (1). In the United States of America, youth e-cigarette use rose dramatically from 1.5% in 2011 to 20.8% in 2018, while the adult (≥18 years) prevalence increased from 3.7% in 2020 to 6.5% in 2023, with the highest rate (15.5%) among those aged 21–24 years (98). In South Korea, the liquid e-cigarette use among adolescents was 3.0% in 2024 (6), while the prevalence of e-cigarette and heated tobacco product use among adults (≥19 years) was 3.5 and 5.9%, respectively, bringing the overall adult tobacco product use to 22.1% (7). Regarding dual use (i.e., concurrent e-cigarette and combustible cigarette use), approximately 30% of American adult e-cigarette users (ECUs) also smoke combustible cigarettes (8), posing greater public health risks than single-product use owing to the increased toxicant exposure and respiratory harm (1, 9).

In response to the global health burden of smoking, the United Nations established Sustainable Development Goal 3.4 aimed at reducing premature mortality from non-communicable diseases by one-third by 2030. SDG 3.a also emphasizes implementation of the World Health Organization’s Framework Convention on Tobacco Control as a core global health strategy (10, 96). In this context, smoking cessation is the most effective intervention for reducing smoking-related health risks (11). For example, even among high-risk combustible cigarette users (CCUs) who smoked more than one pack per day for over 20 years, quitting reduced lung cancer risk by 39% within 5 years, and lowered mortality from cancer and cardiovascular and respiratory diseases by more than 50% after 10 years of abstinence (11, 12). Cessation strategies include pharmacological (e.g., nicotine replacement therapy (NRT), and prescription medications) and non-pharmacological interventions. Some examples of the latter are cognitive behavioral therapy (CBT), motivational interviewing (MI), telephone counseling, and digital programs, helping improve outcomes by enhancing cognitive restructuring, self-efficacy, and social support (13–15). Notably, the bulk of the literature on smoking has focused on pharmacological treatments for both user groups (i.e., CCUs and ECUs) (16), or non-pharmacological interventions primarily targeting CCUs. Considering such foci and the rapid rise of e-cigarette use, we must examine the psychological and behavioral differences between CCUs and ECUs, as related knowledge is essential for the development of appropriate, effective cessation strategies for these distinct groups.

Accordingly, this narrative review compares the commonalities and differences between CCUs and ECUs and proposes directions for optimizing group-tailored non-pharmacological smoking cessation interventions. We focus on non-pharmacological interventions because behavioral mechanisms (e.g., cue-driven habit loops, sensory and identity reinforcement, perceived control) and engagement processes are central to tailoring intervention content and delivery across product types. Although pharmacotherapies remain foundational for many CCUs and some dual users, the rapid growth of digital and behavioral approaches, particularly among ECUs, warrants a focused synthesis of evidence and implementation-relevant intervention components. Here, CCUs are defined as individuals who regularly smoke manufactured or hand-rolled cigarettes, excluding other tobacco products, while ECUs are individuals currently using electronic nicotine delivery systems such as vape pens, pod-based devices, or refillable e-cigarettes. These definitions follow standard classifications used in prior public health and tobacco research (6, 17).

## 2 Materials and methods

This narrative review adopts a descriptive and integrative synthesis approach to summarize representative research trends and derive conceptual implications for clinical practice and public health policy. Unlike systematic reviews, which aim for exhaustive evidence aggregation and quantitative meta-analyses, narrative reviews emphasize conceptual breadth, theoretical integration, and clinical applicability (18). This approach is particularly appropriate for synthesizing data from studies with diverse intervention modalities, heterogeneous populations, and various outcome measures.

We conducted a comprehensive literature search of English-language publications using PubMed, Scopus, Embase, and Google Scholar on November 13, 2025. The search strategy combined user group descriptors (e.g., “combustible cigarette,” “e-cigarette,” “vaping,” and “dual use”) with intervention-related terms (e.g., “smoking cessation,” “cognitive behavioral therapy,” “motivational interviewing,” “digital health,” “quitline,” and “text messaging”). In PubMed, publication date was limited at the day level (2010/01/01–2025/11/13), whereas Scopus and Embase were limited by publication year (2010–2025) owing to platform constraints. For Google Scholar, we screened the first 150 results sorted by relevance, as this database does not support reproducible advanced filtering. Additional sources included Cochrane systematic reviews, the U.S. Clinical Practice Guidelines for Treating Tobacco Use and Dependence, and reports from authoritative health organizations. Additionally, the reference lists of the included studies were manually searched to identify seminal works regardless of publication date. All records were imported into EndNote, where duplicates were removed using automated duplicate detection followed by manual review. The full search terms and results for each database are presented in Supplementary Table S1. Given the review aims, we included diverse study designs. Observational and qualitative studies informed psychological and behavioral profiles (e.g., motivations, perceived harm, and identity/peer processes), while intervention trials and implementation studies informed intervention acceptability, engagement, and cessation-related outcomes. We also included authoritative guidelines and systematic reviews to contextualize evidence strength by intervention category.

Studies were included if they (1) targeted CCUs, ECUs, and/or dual users; (2) evaluated behavioral/digital cessation interventions (with/without adjunct pharmacotherapy); (3) reported user characteristics, intervention acceptability, or behavioral outcomes. Studies were excluded if they (a) focused exclusively on pharmacological interventions without a behavioral/digital cessation component; (b) examined secondhand smoke exposure or population-level policy effects without intervention-level evaluation; (c) were protocols, commentaries, editorials, letters, or conference abstracts without original empirical data; (d) provided insufficient outcome data for synthesis. The inclusion and exclusion criteria used to guide study selection are summarized in Supplementary Table S2.

**Figure 1:**
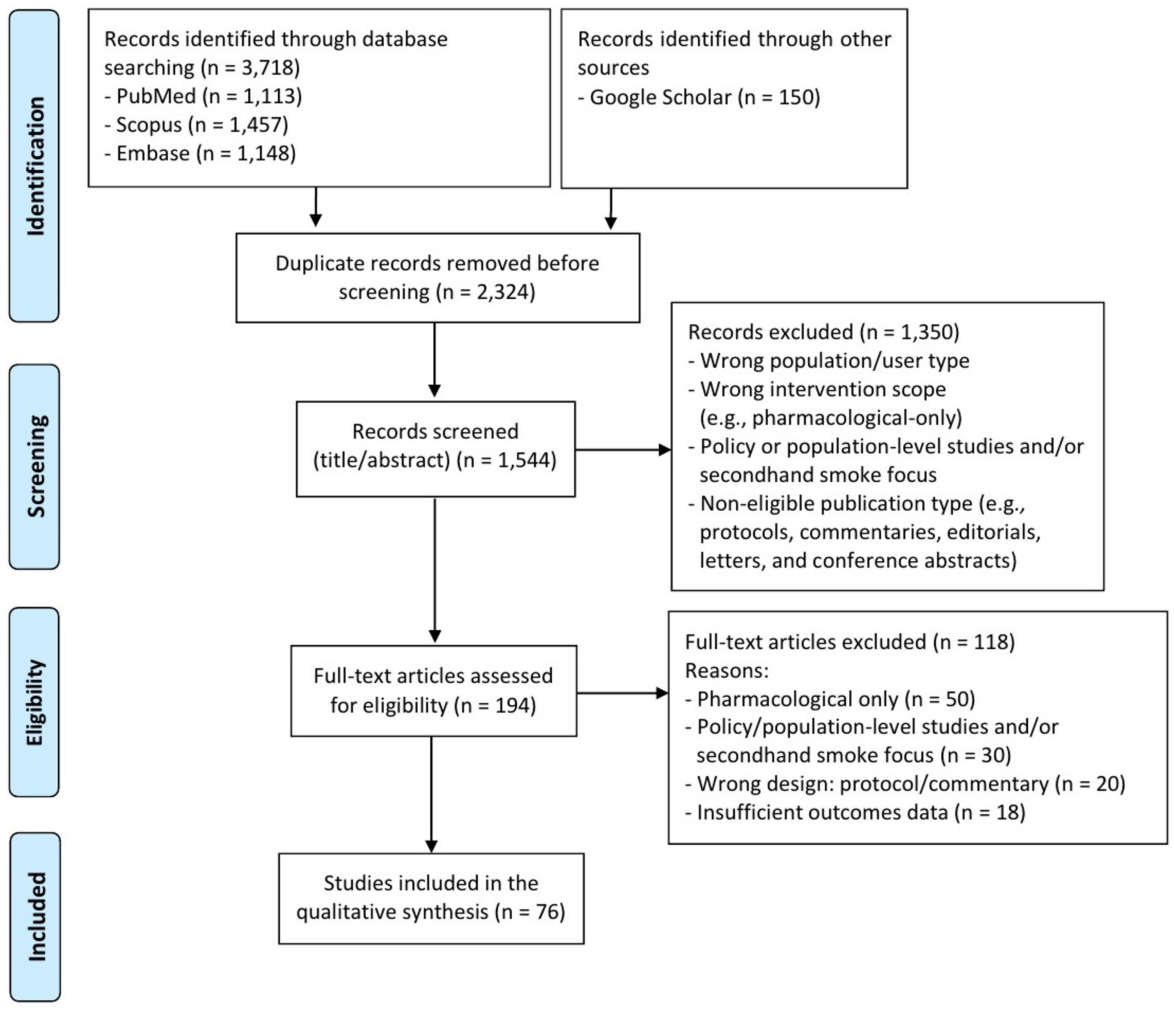
Flow diagram of the study selection process. The diagram summarizes the identification, screening, and inclusion of studies considered in this narrative review. Its structure was adapted from the PRISMA framework to enhance transparency in reporting the literature search and selection process.

During data extraction, we recorded how each study categorized its population and coded samples as exclusive CCUs, exclusive ECUs, dual users (concurrent combustible cigarette and e-cigarette use), or mixed/unclear populations. Importantly, not all studies clearly distinguished between exclusive and dual use; when such distinctions were not reported, findings were classified as mixed/unclear and interpreted accordingly. After study selection, 76 studies were included in the final synthesis. For transparency, the study selection process is summarized in Figure 1 using a PRISMA-adapted flow diagram (97), while acknowledging that the present study follows a narrative review methodology. A detailed overview of the included studies, including study characteristics, populations, interventions, key findings, and their thematic mapping across review sections, is provided in Supplementary Table S3.

The selected studies were narratively synthesized across three thematic domains, as follows: (1) psychological and behavioral profiles of CCUs versus ECUs, (2) evidence supporting non-pharmacological cessation interventions, and (3) comparative effectiveness of interventions across user types. As this study involved a secondary analysis of previously published data, it was exempt from review by the Institutional Review Board of Chung-Ang University Hospital (Exemption No. 2509–016-19594).

## 3 Results

### 3.1 User characteristics by product type

#### 3.1.1 CCUs

Regarding evidence base, this subsection synthesizes findings from three cross-sectional studies (*n* = 3) examining the sociodemographic characteristics of CCUs and patterns of product transition and dual use (7, 19, 20). Many of these datasets include CCUs with e-cigarette experience and do not consistently distinguish exclusive combustible cigarette use from dual use. Therefore, findings should be interpreted as reflecting mixed or unclear cigarette-smoking populations rather than exclusive CCUs. A detailed mapping of the results and discussion sections of contributing studies is provided in Supplementary Table S3.

Within these mixed datasets, CCUs tend to predominantly be middle-aged or older adults and individuals with lower income and educational attainment, often maintaining long-term smoking habits (20) among adults aged ≥ 65 years, smoking prevalence has plateaued or increased, particularly in socioeconomically disadvantaged groups (20). These patterns suggest higher nicotine dependence and more entrenched smoking behaviors, potentially posing greater barriers to cessation.

Regarding transitions to alternative products, data from the U.S. National Adult Tobacco Survey indicate that switching from combustible cigarettes to e-cigarettes increased over time and is more common among younger adults and individuals with lower education and income (7). In contrast, the Korea National Health and Nutrition Examination Survey showed that smokers with e-cigarette experience were younger, more likely to have higher income, and reported stronger quit intentions; among dual users, common reasons for e-cigarette use included perceived cessation support and reduced harm (19). Middle-aged individuals, those with higher socioeconomic status, and certain racial/ethnic groups were less likely to switch, suggesting a persistent preference for combustible tobacco in some subpopulations (19).

Overall, CCUs appear to exhibit entrenched smoking patterns and high nicotine dependence. Standardized cessation approaches may therefore be insufficient, underscoring the need for greater segmentation and tailored intervention strategies for CCUs. We emphasize that these conclusions are based on a limited number of observational studies, may vary by country and measurement, and that the inconsistent differentiation between exclusive combustible cigarette use and dual use in the studies constrains user-type-specific inferences.

#### 3.1.2 ECUs

Regarding evidence base, this subsection synthesizes findings from eight observational studies (cross-sectional, *n* = 7; cohort, *n* = 1) examining determinants of e-cigarette use across different age groups. Studies addressed youth harm perceptions and flavor use (21, 22), peer and social-context influences (23), dual-use motives and dependence patterns (24), and adult motivational profiles, including reduction and cessation intentions (8, 19, 25, 26). Because there is no consistent differentiation between exclusive e-cigarette use and dual use, findings are interpreted according to the most clearly described populations, with mixed samples noted where relevant.

ECUs represent a heterogeneous population, with determinants varying by age and smoking history. Among adolescents and young adults, e-cigarettes are often perceived as less harmful and less addictive than combustible cigarettes (21). Flavored products, particularly the sweet, fruit, mint, and menthol varieties, are highly prevalent at initiation, and disposable devices are commonly used (22). These sensory features are associated with reduced perceived harm and addiction potential and may reinforce continued use. Peer affiliation and social belonging play central roles in adolescent e-cigarette use, with use often occurring in social contexts for inclusion and impression management (23). A substantial proportion of young adult ECUs report no prior combustible cigarette use (8), indicating prevention-relevant initiation pathways and a need for an age-specific interpretation of ECU trajectories.

In adult populations, motivations are more closely linked to smoking history and harm-reduction considerations. Many adults report using e-cigarettes to reduce smoking or support cessation (19, 25). Dual users frequently cite perceived cessation benefits and reduced harm, although convenience and social acceptability are also common motivations (26). Thus, cessation-oriented intent does not necessarily translate into combustible cigarette abstinence, with persistent nicotine use often remaining.

Dual users warrant particular attention. Cross-sectional data highlight smoking reduction, flavor appeal, and indoor-use convenience as common reasons for e-cigarette use, with some users engaging in nicotine self-titration (24). Longitudinal cohort findings show that harm-reduction-oriented motivations do not consistently predict subsequent combustible cigarette abstinence (26), entailing that stated motives may coexist with sustained dual use and elevated total nicotine exposure.

Overall, ECU determinants should be interpreted within age- and smoking-history-specific contexts. Youth use tends to be largely shaped by sensory appeal, perceived reduced harm, and peer influence, whereas adult use tends to more often reflect smoking-related trajectories such as reduction attempts and dual-use maintenance. Distinguishing reported motives from behavioral patterns and total nicotine exposure is essential for informing age-appropriate prevention and cessation strategies.

#### 3.1.3 Psychological and behavioral differences

Regarding evidence base, this subsection integrates evidence from eight sources (cross-sectional, *n* = 5; cohort, *n* = 1; qualitative, *n* = 1; narrative review, *n* = 1) to characterize psychological expectations, perceived control, risk perception, and social-identity processes underlying combustible cigarette use versus e-cigarette use (7, 9, 25–30). Because several studies did not clearly distinguish exclusive e-cigarette use from dual use, user-type-specific conclusions remain limited in parts of this section.

CCUs and ECUs differ not only in product choice but also in psychological expectations, cognitive appraisals, and behavioral regulation strategies (28, 30). These differences extend beyond nicotine delivery method, relating also to mechanisms of dependence formation, risk perception, usage context, and self-regulatory beliefs (Table 1). Among CCUs, smoking behavior is primarily maintained through reinforcement learning processes to avoid nicotine withdrawal. Nicotine functions as a strong primary reinforcer, while sensory cues associated with smoking (e.g., inhalation rituals and smoke) operate as conditioned stimuli. Over time, this process leads to automatized dependence, whereby the withdrawal discomfort and negative affect reinforce continued smoking, particularly during cessation attempts (27, 28). In contrast, ECUs often report a heightened sense of autonomy and control over nicotine use, which is facilitated by the ability to adjust nicotine concentration, device characteristics, and puffing patterns (25, 29). However, this perceived control may reflect sensory expectancy rather than actual dependence reduction, functioning as a cognitive mechanism that legitimizes continued use (28).

**Table 1:** Comparative psychological and behavioral profiles of combustible cigarette and e-cigarette users.

Dimension	Combustible cigarette users	E-cigarette users
Dominant dependence processes	Reinforcement learning aimed at avoiding nicotine withdrawal; conditioned automatism	Nicotine reinforcement remains relevant, with relatively greater salience of sensory expectancy, perceived control, and social-contextual factors
Primary reinforcer	Nicotine as a strong primary reinforcer; sensory cues (e.g., smoke and inhalation) function as conditioned stimuli	Adjustable nicotine concentration, device characteristics, and puffing patterns
Perceived control	Low autonomy; automatized dependence pattern	High perceived autonomy and control, often reflecting sensory expectancy rather than actual reductions in dependence
Risk perception	Generally aware of health risks (e.g., lung disease, cardiovascular disease, and cancer), which motivate cessation attempts	Perceive e-cigarette use as a “cleaner” or “healthier” alternative; frame use as substitution rather than cessation
Role of sensory attributes	Sensory cues maintain habitual use through conditioning	Flavors and vapor aesthetics attenuate perceived risk and negative affect
Social context	Social constraints (e.g., smoke, odor, and public restrictions) serve as external motivators for quitting	Higher social acceptability; integrated into social identity and self-presentation
Social function	Limited; often associated with social stigma	Impression management, peer affiliation, and social gratification (especially among younger users)
Cessation framing	Quitting is perceived as the primary goal	Substitution is used to rationalize continued use

Key implications: these distinctions reflect differences in relative salience and behavioral expression rather than mutually exclusive dependence mechanisms. Cessation interventions should be adapted to the predominant psychological, behavioral, and sociocultural features of each user group.

The two groups also differ regarding risk perception. CCUs are generally aware of smoking-related health risks, which may motivate cessation attempts. By contrast, e-cigarette use is more frequently framed as a cleaner or healthier alternative, and conceptualized as substitution rather than cessation (28, 30). In studies distinguishing exclusive ECUs and dual users, e-cigarette use profiles have been associated with elevated psychological distress compared with never users (9). Moreover, the sensory attributes of e-cigarettes, including flavor and vapor aesthetics, may attenuate perceived risk and reinforce ongoing use (26, 29).

Social meaning further differentiates the two groups. Combustible cigarette use is often constrained by odor and public restrictions, which can serve as external motivators for quitting (7, 26). In contrast, e-cigarette use is more readily integrated into one’s social identity and self-presentation, particularly among younger users, functioning as a medium for peer affiliation and impression management (29).

Overall, combustible cigarette use seems to be more closely linked to withdrawal relief and cue-driven automaticity, whereas e-cigarette use tends to be more strongly associated with sensory expectancy, perceived control, and social identity processes. Tailoring cessation interventions to these distinct psychological, behavioral, and sociocultural profiles may enhance intervention effectiveness. However, most evidence remains observational and varies across populations and measurement approaches, limiting causal inferences.

### 3.2 Non-pharmacological interventions

Regarding evidence base, this section integrates evidence from five sources (systematic review/meta-analysis, *n* = 2; narrative review, *n* = 1; cohort, *n* = 1; quasi-experimental, *n* = 1) to summarize behavioral theory foundations and key non-pharmacological cessation modalities. These include CBT, MI, quitlines, and digital interventions (31–35). Across these sources, the evidence base is primarily derived from CCUs, while evidence specific to ECUs and dual users is more limited—and noted explicitly where applicable.

Non-pharmacological smoking cessation interventions are grounded in multiple complementary behavioral change theories that explain how smoking is initiated, maintained, and ultimately changed (36). The theoretical foundations include learning theory, social cognitive theory, transtheoretical models, and self-determination theory. Learning theory underpins CBT by explaining how smoking behavior develops and is maintained through conditioned cues and reinforcement loops. Social cognitive theory posits that habitual behaviors are governed by outcome expectations, self-efficacy, and observational learning within a social context (37). This theory is foundational for smoking cessation therapeutic strategies focused on enhancing self-efficacy through mastery experiences (e.g., successful coping with cravings) and learning through peer modeling. The transtheoretical model conceptualizes smoking cessation as a process involving sequential stages, which are pre-contemplation, contemplation, preparation, action, and maintenance (38). This model supports the use of stage-matched interventions tailored to individuals’ readiness for change (31). Meanwhile, self-determination theory emphasizes that sustained behavioral change regarding smoking hinges on meeting individuals’ basic psychological needs for autonomy, competence, and relatedness (39). These frameworks collectively inform the design and implementation of various non-pharmacological behavioral interventions for smoking, providing the conceptual basis for evidence-based approaches such as CBT, MI, quitlines, and digital interventions (e-health/mHealth) (32–35).

#### 3.2.1 CBT

Regarding evidence base, this subsection integrates evidence from seven sources (systematic review/meta-analysis, *n* = 3; randomized controlled trial (RCT), *n* = 1; narrative review, *n* = 2; qualitative study, *n* = 1) to summarize core CBT components and effectiveness for smoking cessation, while noting the emerging considerations for adapting CBT-informed approaches to e-cigarette cessation (33, 40–45). Most CBT effectiveness evidence in this subsection comes from CCUs; evidence for e-cigarette cessation remains emergent, being drawn mainly from qualitative or early-stage reports.

CBT, grounded in learning theory and cognitive restructuring principles, aims to identify and modify conditioned cues and maladaptive beliefs associated with smoking. It is a widely used and well-established non-pharmacological intervention for smoking cessation, with its effectiveness supported through systematic reviews and clinical trials (33, 41–43, 46). CBT’s efficacy lies in its multicomponent approach integrating key behavioral and cognitive strategies, with functional analysis (i.e., used to systematically identify the antecedents and consequences that maintain smoking behavior) as a core process. Through functional analysis, clients can recognize specific triggers, thoughts, and emotional states that precede smoking, preparing the groundwork for personalized intervention planning.

CBT’s behavioral techniques focus on disrupting conditioned links, primarily through self-monitoring to identify individual triggers, stimulus control to avoid/minimize cue exposure, and alternative/competing response training to replace smoking with healthy behaviors when cravings occur (47). In terms of cognition, CBT uses cognitive restructuring to identify/challenge the beliefs that justify smoking (e.g., “smoking relieves stress”), helping lower the perceived value of smoking and heighten the client’s self-efficacy (48). Finally, relapse prevention training provides clients with problem-solving, coping, and stress management skills, reframing lapses as opportunities to learn and assisting with the prevention of a full return to smoking (49). CBT is typically delivered in four to ten individual or group sessions using structured tools, including workbooks, behavioral logs, and cognitive restructuring exercises (41, 45).

CBT’s effectiveness in reducing cigarette consumption in the short-term has been demonstrated, even among smokers with moderate nicotine dependence who are not willing to quit, and it has been reported to support relapse-prevention strategies during the early stages of smoking cessation (33, 43). Cochrane reviews rate CBT as one of the most consistently effective behavioral interventions (42), while American clinical guidelines recommend CBT as an effective standalone treatment (46). However, in individuals with high nicotine dependence or severe withdrawal symptoms, cessation intervention outcomes may be strengthened through the combination of CBT with NRT or pharmacotherapy (40, 41). A multimodal approach that integrates CBT with MI or NRT may offer synergistic benefits by addressing both motivational and behavioral components. Moreover, group-based CBT has shown particular benefits for specific populations (33), such as African American and Hispanic CCUs, although individual therapy may yield higher quit rates than group formats among CCUs (41).

For ECUs, CBT interventions are still nascent. Their distinct motivational profiles, behavioral patterns, and nicotine use characteristics require tailored CBT approaches that can adequately address the specific needs of this growing group (44).

#### 3.2.2 MI

Regarding evidence base, this subsection integrates evidence from six sources (systematic review/meta-analysis, *n* = 2; RCT, *n* = 1; cohort, *n* = 2; qualitative study, *n* = 1) to summarize MI techniques and effectiveness for smoking cessation and implementation in clinical and quitline settings (14, 50–54). MI evidence is predominantly drawn from CCUs, while evidence involving dual users or ECUs is limited; where samples are mixed or user groups are not clearly distinguished, this is noted as a limitation.

MI draws primarily on self-determination theory and the transtheoretical model, emphasizing intrinsic motivation and readiness to change to assist individuals with resolving ambivalence and enhancing self-efficacy through empathic, collaborative communication rather than directive advice. It is particularly effective for individuals lacking in intrinsic motivation or exhibiting ambivalence about behavioral change (55). MI uses empathic counseling techniques that avoid providing direct advice to allow individuals to explore their own reasons and values for change, thereby enhancing self-determined motivation and self-efficacy (50, 54).

The core communication techniques in MI are open-ended questions, affirmations, reflective listening, and summaries, which facilitate self-exploration and insight (55). It also relies on key strategies such as expressing empathy and developing discrepancy, through which clients are taught to see the mismatch between their current smoking behavior and personal goals/values. A critical element is “rolling with resistance,” referring to when clients show reluctance or push back and the counselor accepts this resistance without argument, gently redirecting the dialogue such as to use resistance as momentum for change. The notion is that supporting self-efficacy by highlighting the client’s confidence and ability to quit further strengthens motivation. The use of the 5R’s (i.e., relevance, risks, rewards, roadblocks, and repetition) provides a structured framework for exploring and enhancing motivation in a respectful, non-confrontational manner, which is essential for effective smoking cessation counseling.

MI has demonstrated efficacy as a standalone intervention for smoking cessation, with multiple RTCs and meta-analyses reporting significantly higher quit rates than usual care or brief advice, particularly in short-term outcomes (53, 54). It may be particularly relevant for adolescents, dual users, and ECUs who often have a low perceived need for cessation or limited cessation attempts (14, 51).

However, for CCUs with high nicotine dependence, repeated quit failures, and prominent withdrawal symptoms, MI alone appears insufficient (52–54). Although it effectively enhances motivation in the precontemplation and contemplation stages, sustained abstinence and relapse prevention generally require additional behavioral strategies or pharmacotherapy (46, 53, 54). Among ECUs, comparable findings have been reported: MI-based interventions increased initial quitting motivation and reduced consumption among adolescents and young adults, but long-term maintenance often necessitated adjunctive approaches such as NRT or strategies to counter peer influence (56).

The clinical guidelines recommend combining MI with NRT or CBT as part of a multicomponent approach. Although MI alone may be effective in adolescent CCUs or CCUs with lower nicotine dependence, in the context of diverse populations, the likelihood for sustained cessation to be achieved is greater when using tailored and integrated interventions (14, 50, 51). Although MI is typically delivered in individual sessions, group-based formats have been explored for alcohol and drug use (57) and obesity management (58). However, empirical evidence specific to smoking cessation remains limited.

#### 3.2.3 Quitlines and text messaging interventions

Regarding evidence base, this subsection integrates evidence from 10 sources (systematic review/meta-analysis, *n* = 2; RCT, *n* = 7; quasi-experimental, *n* = 1) to summarize quitline- and SMS-based cessation interventions, including telephone/video counseling delivery and culturally tailored text messaging programs (35, 59–67). Evidence for quitlines and SMS interventions is more abundant for CCUs, with a smaller but growing RCT evidence base for e-cigarette cessation among ECUs; user group is specified when describing each intervention effect.

Counselor-delivered quitlines and mobile-based text messaging interventions are widely used non-pharmacological strategies for smoking cessation owing to their effectiveness in improving treatment adherence and abstinence retention (35, 63, 66). The effectiveness of quitlines hinges on strengthening the motivation to quit and providing behavioral strategies through trained counselors, particularly in settings with limited access to face-to-face care (61, 66, 68). According to a Cochrane systematic review, quitline services significantly increased smoking cessation likelihood—by 1.38 times among CCUs who voluntarily enrolled and by 1.25 times in the overall recruited population—and were effective both as single-session and repeated counseling interventions (66). Recently, quitline-based interventions have expanded to real-time video counseling, offering an innovative modality that enables the delivery of evidence-based techniques (e.g., CBT and MI) while incorporating nonverbal interaction (60, 69).

As mentioned above, quitlines rely on trained counselors, while text messaging interventions deliver scalable, automated, and continuous support to large populations. The literature shows the significant effectiveness of quitlines in both its single-session and repeated counseling formats (66), and text messaging interventions achieve similar behavioral reinforcement through frequent and automated messaging. They are especially effective among adolescents, young adults, and digitally connected groups, with their low development and implementation costs making them attractive, cost-effective strategies for implementation in healthcare systems. For example, in a RCT among CCUs, TXT2STOP achieved a six-month biochemically verified abstinence rate of 10.7% in the intervention group compared with 4.9% in controls, corresponding to a relative risk of 2.20 (95% CI, 1.80–2.68) (63). Among ECUs, This is Quitting nearly doubled the 30-day abstinence rate at 1 month compared to controls, with effects maintained at longer follow-ups in RCTs (64, 65). Text messaging interventions have also been effective among disadvantaged and minority populations, where theory-driven designs have demonstrated enhanced outcomes (35, 62). For example, among Spanish-speaking Latino CCUs, a culturally tailored program achieved high acceptability and engagement (62), while Text2Quit improved 6-month abstinence to 11.1% (vs. 5.0% in a self-help control group) (59).

Overall, quitlines rely on human interaction to support adherence and relapse prevention, while text messaging interventions highlight the potential of culturally adapted, theory-based, and cost-effective strategies to achieve clinically meaningful cessation outcomes. They function as complementary approaches, one grounded in intensive interpersonal counseling and the other in scalable, automated message delivery, that can together offer flexible and practical strategies applicable from adolescent ECUs to socioeconomically disadvantaged CCUs (35, 64, 65, 67, 99).

#### 3.2.4 Digital health-based interventions

Regarding evidence base, this subsection includes 15 studies (RCT, *n* = 7; systematic review/meta-analysis, *n* = 1; cohort, *n* = 3; cross-sectional, *n* = 2; quasi-experimental, *n* = 1; narrative review, *n* = 1) (70–84). Digital intervention evidence differs by user group: robust efficacy data are available for CCUs, whereas evidence for e-cigarette cessation among ECUs and prevention among youth is more heterogeneous and sometimes based on mixed/unclear samples, which is noted where relevant.

The rapid expansion of smartphones, applications, and telehealth platforms has transformed the smoking cessation landscape. Digital health-based smoking cessation interventions include mobile apps, web-based programs, gamification, AI-powered chatbots, virtual reality (VR), and wearable technologies, all offering high accessibility and scalability and allowing personalized, user-centered interventions (77, 85). These smartphone-based cessation apps may be well-suited for digitally engaged populations, with features such as gamification, real-time feedback, and social interaction enhancing engagement and adherence (85). Unlike traditional approaches, digital interventions can collect real-time behavioral data and deliver tailored CBT content and feedback based on user motivation and usage patterns, although the evidence on effectiveness and optimal approaches diverges substantially by smoking group. For CCUs, CBT-based digital interventions have demonstrated strong efficacy in multiple large-scale studies; the Quit Genius platform, which combines smartphone-delivered CBT content with one-on-one coaching, showed significantly superior quit rates compared to brief advice interventions in a RCT in the UK, with benefits sustained at a 1-year follow-up (82, 83). The results were similar for other cultural contexts, including a Chinese study that found substantially higher continuous abstinence rates with CBT-based smartphone applications (vs. control conditions) (70). CCU-focused multi-component integrated programs combining multiple evidence-based elements have shown particular promise: The Pivot program integrates personal carbon monoxide breath sensors, smartphone applications, in-app messaging–based coaching, and NRT, demonstrating strong engagement and clinically meaningful abstinence outcomes in cohort studies (75). The CureApp Smoking Cessation system, tested in Japan, combines smartphone apps with web-based patient management and mobile carbon monoxide monitoring, achieving significantly higher and biochemically verified continuous abstinence rates that were sustained over extended follow-up periods (78).

Another core strategy is gamification, using elements such as scoring, challenges, goal setting, and visual progress tracking to enhance self-efficacy and motivation (73). Gamified smoking cessation apps have demonstrated positive correlations between game-element engagement and quitting success, with level achievement and progress markers being associated with improved abstinence rates (80). AI chatbot integration also shows considerable promise, as chatbot-enhanced apps can significantly increase user engagement compared with standard app versions (79).

For ECUs, digital interventions represent an emerging field with distinct approaches focused on two primary populations: adults seeking cessation and adolescents requiring prevention. Cessation interventions for adult ECUs have adopted successful approaches from traditional smoking cessation, such as the aforementioned Pivot program. When modified for e-cigarette cessation, the program demonstrated high user engagement and clinically meaningful abstinence outcomes (76). Acceptance and Commitment Therapy-based interventions have also shown preliminary efficacy in pilot studies with young adult ECUs, demonstrating improved quitting attempt rates and modest abstinence outcomes (72). Prevention interventions for adolescent ECUs have leveraged innovative technologies, particularly virtual reality (VR) and gaming platforms. VR-based prevention games have shown significant improvements in e-cigarette use knowledge and harm perceptions while reducing future e-cigarette use intentions among middle school students (84). Gaming platforms familiar to youth, such as Minecraft-based prevention interventions, have achieved substantial reach and engagement and demonstrated particular promise for preventing initiation among non-users (74).

Evidence reveals distinct maturation patterns for interventions focused on CCUs and ECUs (76, 82). For CCUs, multicomponent interventions combining CBT, sensor technology, and professional coaching feature the strongest evidence base, with multiple large-scale RCTs supporting their effectiveness (78, 83). Theoretical frameworks and validated measurement approaches have also been developed in this field. For ECUs, the research landscape remains smaller in scale, with prevention interventions showing promise for knowledge enhancement and attitude change and cessation interventions demonstrating feasibility but requiring larger validation studies (72, 74, 84).

Engagement remains a critical challenge in both populations, as most digital interventions have shown high dropout rates. However, the integration of objective measurement tools, AI enhancement, and personalized content delivery shows promise for improving sustained engagement (78). Future research priorities include developing standardized outcome evaluation methods, establishing validated digital interventions specifically for ECUs, and devising optimal integration pathways with healthcare systems for both populations (73, 76). Machine learning-based enhanced personalization represents a promising frontier for improving intervention effectiveness across both the CCU and ECU populations (71, 81).

### 3.3 Comparative effectiveness by user type

#### 3.3.1 CCUs

Regarding evidence base, this section integrates evidence from 13 sources (systematic review/meta-analysis, *n* = 7; RCT, *n* = 4; cohort, *n* = 1; narrative review, *n* = 1) to summarize the comparative effectiveness of non-pharmacological cessation approaches for CCUs and subgroup considerations, including counselling/CBT-oriented support, MI, and text-messaging interventions (14, 32, 33, 35, 41, 42, 51, 54, 59, 63, 86–88). The comparative effectiveness evidence in this subsection is primarily drawn from CCUs, such that findings are interpreted as being applicable to CCUs unless otherwise specified. Non-pharmacological smoking cessation interventions for CCUs have been extensively studied and shown relatively consistent patterns of effectiveness (32). Structured and multicomponent approaches have demonstrated significant benefits (46).

##### 3.3.1.1 CBT’S effectiveness

In CCUs, CBT has shown superior cessation outcomes compared to control conditions, with particular strengths in coping with high-risk situations and preventing relapse (42). Systematic reviews confirm its consistent efficacy, which is enhanced when combined with NRT (46). Through cognitive restructuring and coping skills training, CBT effectively addresses strong nicotine dependence and automated smoking patterns, supporting long-term abstinence (33, 41).

##### 3.3.1.2 MI’s effectiveness and limitations

MI is effective in initiating motivation in CCUs with low readiness to quit or ambivalence (14, 54). However, its exclusive use tends to reduce efficacy during long-term follow-up, with optimal outcomes observed when combined with structured behavioral interventions or pharmacotherapy (46, 51).

##### 3.3.1.3 Effectiveness of quitlines and text messaging interventions

Quitlines and text messaging programs provide strong advantages regarding accessibility and cost-effectiveness, with repeated evidence of improved cessation outcomes compared with standard care (32, 35). These effects are particularly pronounced among voluntary participants, and frequent automated messages have been shown to support daily motivation and relapse prevention (59, 63).

##### 3.3.1.4 Subgroup differences and the need for tailored approaches

Intervention responsiveness varies across subgroups according to age, socioeconomic status, and dependence level (33). Some studies reported higher engagement and effectiveness of group-based CBT, while others found that individual-based CBT was more suitable for certain populations (41). Older adults often show greater acceptance of face-to-face or telephone counseling, whereas middle-aged groups respond more positively to structured CBT or MI combined with pharmacotherapy (46, 66, 87, 88).

#### 3.3.2 ECUs

Regarding evidence base, this section integrates evidence from eight studies (RCT, *n* = 1; cohort, *n* = 2; cross-sectional, *n* = 2; qualitative, *n* = 1; quasi-experimental, *n* = 1; case series, *n* = 1) to summarize non-pharmacological cessation and prevention-relevant determinants for ECUs, including digital/mHealth programs, telehealth contingency management, perceived barriers, and social influence factors (21, 23, 26, 34, 44, 56, 76, 89). Across these studies, evidence specific to ECUs remains limited and heterogeneous; where findings are drawn from dual-user or mixed/unclear samples, this is stated explicitly and interpreted with caution. Research on non-pharmacological cessation interventions for ECUs is still nascent. Nevertheless, many studies have highlighted the need for strategies distinct from those applied for CCUs (35, 44).

##### 3.3.2.1 Effectiveness of digital-based interventions

Higher engagement and feasibility have been reported in app- and platform-based e-cigarette cessation interventions among ECUs, particularly those incorporating real-time feedback and self-monitoring features (34). Adult-focused app-based programs also report high retention and clinical improvements (e.g., reduced consumption and dependence), suggesting potential value for digitally delivered, user-centered cessation support (34, 76). Given the limited validity of carbon monoxide in the context of ECUs, alternative measures (e.g., urinary/salivary cotinine and device-derived use metrics) may be considered for outcome assessment and tailoring; carbon monoxide may still be informative for verifying combustible cigarette reduction or cessation among dual users.

##### 3.3.2.2 Importance of stepwise goal-setting

ECUs show greater acceptance and engagement when interventions emphasize stepwise goals (e.g., reduction or switching) over abrupt cessation (44, 89). This group may be more prone to accept framing goals around gradual change (44).

##### 3.3.2.3 MI’s potentials and limitations

MI-informed components are commonly incorporated into e-cigarette cessation program designs for adolescents and young adults with low readiness or ambivalence (44, 56). However, its long-term impact is limited, and integration with NRT, peer-influence management, or digital coaching is recommended (56).

##### 3.3.2.4 Peer influence and social factors

Peer norms and social belonging strongly shape e-cigarette use among adolescents, making peer norm modification and alternative social activities essential intervention components (23). Adolescents’ strong preferences for flavors and some sensory features of e-cigarettes reduce harm perception and reinforce use, suggesting that sensory substitution and cognitive reframing should be emphasized (21). Relatedly, among dual users, convenience and social acceptability motives may reinforce continued e-cigarette use alongside combustible cigarette use (26).

##### 3.3.2.5 Differentiated intensity and delivery approaches

ECUs may respond to low-intensity, intermittent interventions; however, sustained engagement and motivation may be better supported by app- and platform-based designs incorporating gamification, social functions, and real-time feedback (34, 89). Mobile programs integrating NRT, asynchronous coaching, and self-directed CBT content can leverage ECUs’ digital affinity and autonomy, potentially yielding favorable short-term cessation outcomes (34). Table 2 summarizes the comparative effectiveness of non-pharmacological interventions in CCUs and ECUs.

**Table 2:** Comparison of non-pharmacological smoking cessation interventions for combustible cigarette users and e-cigarette users.

Intervention	Theoretical basis	Core techniques	Effectiveness for combustible cigarette users	Effectiveness for e-cigarette users
CBT	Learning theory, cognitive restructuring	• Functional analysis • Self-monitoring • Stimulus control • Alternative response training • Cognitive restructuring • Relapse prevention	• Well-established efficacy in systematic reviews • Effective for short-term reduction • Recommended as a standalone treatment • Combination with NRT/pharmacotherapy for high dependence cases	• Nascent evidence • Tailored approaches needed for ECU-specific profiles
MI	Self-determination theory, transtheoretical model	• OARS: Open questions, Affirmations, Reflective listening, Summaries • Expressing empathy • Developing discrepancy • Rolling with resistance • Supporting self-efficacy • 5R’s framework	• Effective as a standalone intervention • Suitable for the precontemplation/contemplation stages • Insufficient when used in isolation for high dependence • Combination with NRT/CBT recommended	• Low perceived need for cessation • Particularly relevant for adolescents, dual users, and ECUs • Increased initial quitting motivation and reduced consumption • Effect maintenance requires adjunctive approaches
Quitlines	Counselor-delivered behavioral strategies	• Telephone counseling by trained counselors • Motivation enhancement • Behavioral strategy delivery • Video counseling	• Significantly increased cessation likelihood • Effective in single and repeated sessions • Useful in limited face-to-face access settings	• Limited ECU-specific research
Text messaging interventions	Automated scalable behavioral reinforcement	• Automated frequent messaging • Cultural and linguistic tailoring • Theory-based design	• Significantly higher abstinence rates vs. controls • Effective in disadvantaged/minority populations • Cost-effective health system strategy	• Nearly doubled 30-day abstinence rate (1-month; ECUs) • Effects maintained at longer follow-up • Effective for adolescents and young adults
Digital healthcare-based interventions	Multi-component integration, personalization, real-time feedback	• Mobile apps and web programs • Gamification • AI chatbots • VR • Wearables (CO sensors)	• CBT-based apps show superior quit rates • Multi-component programs demonstrate strong efficacy • Gamification enhances engagement and outcomes • AI chatbots increase user engagement	• Emerging field with smaller-scale studies • Cessation evidence still limited, while that on prevention is stronger • Preliminary evidence: high engagement and meaningful abstinence outcomes • Improves knowledge and harm perceptions • Promising for preventing initiation

CBT, cognitive behavioral therapy; CCUs, combustible cigarette users; CO, carbon monoxide; ECUs, e-cigarette users; MI, motivational interview; NRT, nicotine replacement therapy; VR, virtual reality.

## 4 Discussion

### 4.1 Key findings

Our results suggest that CCUs and ECUs may represent distinct populations with differing motivational structures, dependence patterns, and behavioral characteristics. CCUs in the reviewed literature are often characterized by middle-aged and older adults with established nicotine dependence, and may respond more favorably to structured, higher-intensity interventions such as CBT combined with MI or pharmacotherapy. In this group, cessation processes are often driven by physiological withdrawal management and conditioned response disruptions. In contrast, ECU samples span adolescents, young adults, and adults, and are often described as showing profiles characterized by perceived control, social identity considerations, and sensory expectancies rather than traditional physical dependence patterns.

**Figure 2:**
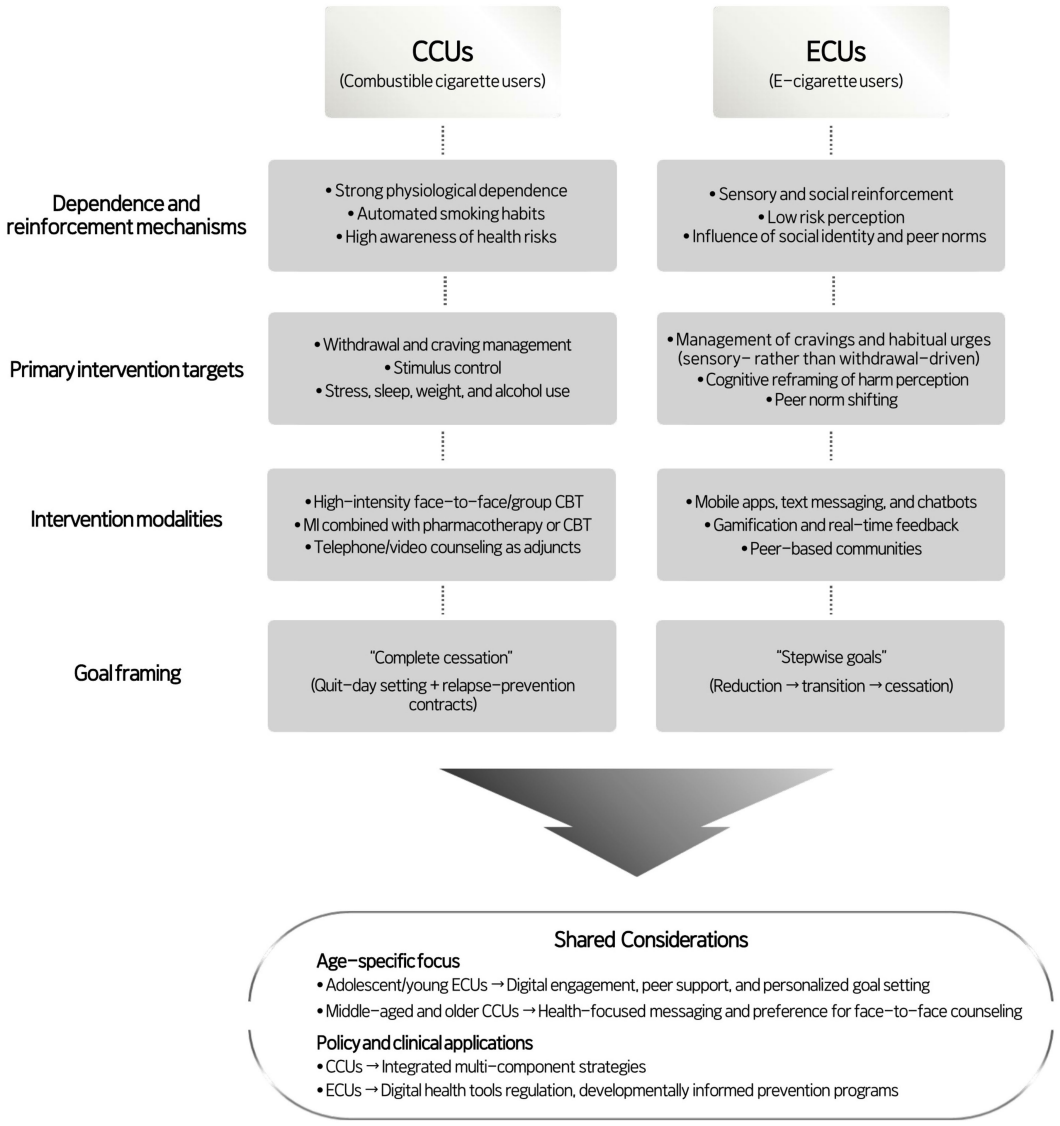
Conceptual framework for tailored non-pharmacological smoking cessation interventions. CBT, cognitive behavioral therapy; MI, motivational interviewing.

The reviewed evidence suggests differential patterns of effectiveness and engagement across these user types. For CCUs, traditional evidence-based approaches are supported by a more established evidence base and generally demonstrate efficacy, with multicomponent strategies yielding superior outcomes. For ECUs, extant studies suggest that engagement and short-term outcomes may be more favorable in digitally delivered interventions encompassing mobile apps with gamification elements, real-time feedback systems, and peer-oriented social components. Our synthesis suggests that ECUs may benefit from flexible, user-controlled interventions that accommodate their preferences for gradual reduction over immediate, complete cessation. These differential pathways are empirically grounded in prior evidence demonstrating that the withdrawal-driven dependence and conditioned smoking automatism among CCUs respond best to structured, high-intensity, multicomponent behavioral interventions; meanwhile, the sensory reinforcement, perceived control, and peer-norm influences among ECUs are associated with higher engagement in digitally delivered, socially embedded, and stepwise interventions. Figure 2 summarizes the behavioral conceptual framework linking user characteristics to tailored intervention strategies.

### 4.2 Framework derivation and theoretical grounding

Regarding evidence base, this subsection integrates evidence from five sources (systematic review/meta-analysis, *n* = 1; cross-sectional, *n* = 1; qualitative study, *n* = 2; narrative review, *n* = 1) to support the inductive derivation of the differentiated behavioral conceptual framework for CCUs and ECUs (27–29, 32, 44). The differentiated behavioral conceptual framework proposed in this review was inductively derived from convergent patterns observed across the synthesized literature on (1) product-specific dependence mechanisms, (2) psychological and social reinforcement processes, and (3) differential responsiveness to non-pharmacological cessation interventions among CCUs and ECUs.

Across multiple studies, CCUs were characterized by withdrawal-driven nicotine dependence and conditioned, automated smoking habits (27), which were most effectively addressed by structured, high-intensity, multicomponent interventions (32, 46). Conversely, ECUs were more strongly influenced by sensory reinforcement, perceived behavioral control, and peer norms (28, 29), with higher engagement observed for digitally delivered, socially embedded, and stepwise interventions (44) (Graham et al., 2020). Integrating these recurring empirical patterns, the framework links user profiles to theoretically aligned intervention targets, delivery modalities, and goal-framing strategies and is intended to guide hypothesis-driven intervention design and future comparative effectiveness research. This integrative conceptual framework is schematically summarized in Figure 2.

### 4.3 Clinical implications for tailored interventions

#### 4.3.1 User-specific intervention design

Regarding evidence base, this section integrates evidence from nine sources (systematic review/meta-analysis, *n* = 3; RCT, *n* = 1; cohort, *n* = 1; qualitative, *n* = 1; narrative review, *n* = 2; quasi-experimental, *n* = 1) to inform user-specific intervention designs for CCUs and ECUs, spanning counselling/CBT-oriented behavioral support, MI-related motivation findings, and digitally delivered cessation approaches (14, 33–35, 40, 44, 45, 68, 76).

##### 4.3.1.1 Structured multi-component approaches for CCUs

Given the high nicotine dependence and automatic smoking patterns of CCUs, structured high-intensity interventions with multiple sessions and pharmacotherapy are recommended (46, 68). MI is useful for initiating motivation, but sustained cessation is best achieved when linked with CBT and pharmacotherapy (14, 46). Particularly during the early stages of smoking cessation, active behavioral techniques must be implemented focusing on disrupting the conditioned links, primarily through self-monitoring to identify individual triggers, stimulus control to avoid/minimize cue exposure, and alternative/competing response training to replace smoking with healthy behaviors when cravings occur (47). Integrating systematic strategies for withdrawal symptom and relapse trigger (e.g., stress, sleep, weight concerns, and alcohol use) management and providing intensive follow-up during the first weeks of cessation are critical for long-term success (33, 46). Multicomponent approaches such as CBT + MI, CBT + NRT, or triple combinations (CBT + MI + NRT) consistently show superior outcomes compared to single interventions (45, 68).

##### 4.3.1.2 Flexible and engaging approaches for ECUs

ECUs are driven less by physiological withdrawal and more by a perceived sense of control, sensory gratification, and social identity. Their usage is often framed as self-regulation (e.g., “I can manage my nicotine use”), which paradoxically sustains dependence. Therefore, non-pharmacological interventions tailored to ECUs should directly target these unique psychological mechanisms. First, cognitive restructuring should focus on correcting the illusion of control and helping users recognize that perceived autonomy over device use or nicotine concentration often reinforces dependence. Second, sensory substitution techniques (e.g., deep breathing, menthol lozenges, and tactile grounding) can serve as behavioral replacements for inhalation rituals and address the sensory expectations that maintain use. Third, peer- and identity-based motivational strategies are essential, particularly for adolescents and young adults; group-based MI or digital social challenges can reframe e-cigarette cessation as a symbol of autonomy and health-oriented self-expression, rather than social inclusion.

ECUs demonstrate high digital affinity and autonomy, showing greater engagement and effectiveness with app- and platform-based interventions that provide low-intensity but continuous monitoring and feedback (35). Allowing users to self-regulate their goals and pace, combined with the provision of personalized feedback and contingency reinforcement, enhances sustainability and acceptability (40, 44). Moreover, modular CBT delivered through applications, chatbots, or remote coaching, when integrated with real-time feedback from wearables or app−/device-derived use metrics—and, where relevant, biochemical verification such as cotinine—may support behavioral change and relapse prevention (34, 76).

#### 4.3.2 Transitional interventions for dual users

Regarding evidence base, this section integrates evidence from five sources (qualitative, *n* = 1; RCT, *n* = 3; cross-sectional, *n* = 1) to inform transitional intervention components for dual users, including perceived adaptation challenges, quitline-based coaching, and digitally delivered monitoring/support elements (65, 90–93).

Many smokers transitioning from combustible cigarettes to e-cigarettes experience early frustration owing to initial nicotine delivery differences and sensory mismatches, which can contribute to dual use or relapse. Among dual users, concurrent access to both products can sustain or increase total nicotine intake and complicate dependence management, because nicotine delivery, cues, and use contexts may reinforce one another rather than promoting a full transition to e-cigarettes. To address these challenges of dual users, a phased transitional intervention model that integrates physiological, cognitive, and behavioral components is recommended.

Dual users also exhibit heterogeneous motivational profiles: some use e-cigarettes as a deliberate step toward cigarette cessation, whereas others maintain e-cigarette use for flexibility, convenience, or situational acceptability, which can prolong concurrent use if the goals are not explicitly negotiated. Accordingly, transitional interventions should be dual-targeted—explicitly addressing both combustible cigarette and e-cigarette use (i.e., product-specific triggers, tapering/quit plans, and coordinated pharmacotherapy pathways)—rather than applying a single-product cessation framework.

First, physiological calibration helps users manage nicotine self-titration and reduces withdrawal discomfort. Short-term NRT and guidance for adjusting e-liquid concentrations can stabilize nicotine intake (91, 92). Second, cognitive reframing assists users in interpreting early discomfort as a normal adaptation rather than failure, using MI to clarify the functional role of smoking (e.g., stress relief) and introduce non-nicotine coping strategies such as deep breathing and mindfulness (93). Third, behavioral stabilization can be supported through digital monitoring tools and personalized feedback to track cigarette consumption and reinforce self-efficacy (65, 90).

These interventions frame the CCU–ECU transition not as relapse but as a temporary adaptive stage in the cessation continuum. Transitional approaches may facilitate sustained cessation and prevent prolonged dual use if they combine physiological support with cognitive and behavioral reinforcement. A key implementation priority is preventing “stable dual use” by setting explicit product-specific targets and monitoring progress in both behaviors over time.

##### 4.3.2.1 Age-specific tailoring strategies

Regarding evidence base, this section integrates evidence from nine sources (systematic review/meta-analysis, *n* = 3; RCT, *n* = 3; cohort/feasibility, *n* = 2; qualitative, *n* = 1) to summarize age-specific tailoring considerations for ECUs and CCUs, explicitly distinguishing adolescents, young adults (18–25), and adults (>25) where relevant (34, 35, 44, 64, 65, 69, 76, 86, 89).

##### 4.3.2.2 Adolescent ECUs (13–17 years)

In adolescents, e-cigarette use is strongly shaped by social-context drivers and peer norms, indicating that tailoring should explicitly address social belonging and normative influences (44). Text-messaging cessation programs have demonstrated improved abstinence outcomes among adolescent ECUs compared with assessment-only controls (65). Because e-cigarette use is often convenient and discreet, intervention content should also target consumption awareness and reduce automatic use patterns in daily contexts (44). Mobile-delivered digital support, including automated text messaging, can provide scalable and continuous reinforcement and has shown cessation benefits (vs. minimal support) (35).

##### 4.3.2.3 Young adult ECUs (18–25 years)

Among young adults, acceptability may improve when interventions strengthen autonomy and perceived control through self-monitoring and personalized goal setting (44). Providing readily accessible mobile or remote support is a realistic strategy for this group, and SMS/text-messaging cessation programs have demonstrated improved abstinence outcomes in young adult ECUs (64). In a small pilot trial, telehealth-delivered, app-based contingency management showed high feasibility and acceptability among young adult ECUs and increased the proportion of abstinent cotinine samples during treatment (vs. monitoring controls), although end-of-treatment and follow-up abstinence differences were not statistically significant (89). App-based programs that incorporate real-time feedback and other engagement features may support sustained participation and use reduction (76).

##### 4.3.2.4 Adult ECUs (>25 years)

For adults (>25 years), e-cigarette cessation support may be most acceptable when it acknowledges autonomy and pragmatic harm-reduction intentions while defining stepwise targets toward nicotine discontinuation (34, 44). Given e-cigarette-specific barriers such as convenience/discreet use and limited awareness of consumption, structured self-monitoring and personalized feedback delivered via digital channels may be particularly relevant (34, 44). Where clinically appropriate, pharmacotherapy (e.g., NRT) integration with behavioral support can strengthen cessation outcomes (86). In adult ECUs, feasibility evidence supports NRT implementation within digitally delivered behavioral programs (34).

##### 4.3.2.5 Middle-aged and older adult CCUs (≥35 years)

Middle-aged and older adult CCUs often benefit from structured professional behavioral support, particularly when delivered with pharmacotherapy (86). Telephone and video counseling can improve accessibility and have demonstrated comparable effectiveness to face-to-face care in available evidence, supporting integration into routine practice for this demographic (69).

#### 4.3.3 Differentiation of intervention content and delivery

Regarding evidence base, this section integrates evidence from 12 sources (systematic review/meta-analysis, *n* = 4; RCT, *n* = 3; cohort, *n* = 1; quasi-experimental, *n* = 1; qualitative, *n* = 1; cross-sectional, *n* = 2) to differentiate goal framing, intervention intensity, and technology-enabled delivery for CCUs versus ECUs (19, 24, 34, 35, 41, 44, 45, 59, 69, 76, 89, 94).

##### 4.3.3.1 Goal-setting and framing

Given their high dependence and withdrawal severity, CCUs should receive structured interventions (CBT, MI, and pharmacotherapy) that emphasize complete cessation as the ultimate goal, with quit dates, behavioral contracts, and relapse-prevention plans being established in advance (46, 66). Framing cessation through “complete abstinence” rather than gradual reduction enhances motivation and facilitates rapid re-engagement after relapse (41, 94).

For ECUs, stepwise goals such as reducing use frequency, nicotine concentration, and triggers, progressing to nicotine-free products, and ultimately suggesting cessation, are more realistic (44). Among adolescents and young adults, combining sensory substitution, peer norm shifts, and gradual cognitive reframing improves acceptability and adherence, as proposed by the transtheoretical model and its principle of incremental goal-setting (38, 76, 89).

##### 4.3.3.2 Intensity and frequency of intervention

For CCUs, high-intensity structured sessions delivered over several weeks, combined with follow-ups at 3, 6, and 12 months, improve long-term abstinence rates (45, 46). For ECUs, they may prefer low-intensity but sustained interventions with on-demand support and frequent micro-interventions delivered via text messages or apps (34, 35).

##### 4.3.3.3 Technology use and accessibility

For CCUs, text-based support serves as a practical adjunct, while the groundwork should be laid by face-to-face and telephone counseling (59, 86). For ECUs, digitally intensive designs are central and should incorporate mobile apps, remote coaching, video counseling, and wearable integration while focusing on real-time feedback and personalized messaging (69, 76). The key intervention strategies tailored to CCUs and ECUs are summarized in Table 3.

**Table 3:** Tailored intervention strategies for combustible cigarette users and e-cigarette users.

Domain	Combustible cigarette users	E-cigarette Users
Target mechanisms	Withdrawal and craving management; integration of relapse triggers (stress, sleep, weight, and alcohol)	Shift from sensory/social reinforcement; cognitive reframing of risk perception
Delivery medium and intensity	Face-to-face/group CBT with pharmacotherapy; telephone and video counseling as adjuncts	Apps, chatbots, and wearable integration (platform-based); brief and frequent interventions; SMS/text messaging (channel-based); scalable, automated reinforcement
Goal framing	Clear ultimate goal of “complete cessation,” with quit-day setting and relapse prevention contracts	Stepwise goals (reduction → transition → cessation)
Clinical application	Integrated pathways across primary care–mental health–community; high-intensity multi-component strategies	Digital- and peer-based intervention packages; self-directed structures
Policy implications	Strengthening the professional workforce for middle-aged and older adult CCUs	Prevention and intervention for adolescent/young adult ECUs; regulation and certification systems for digital health tools

CBT, cognitive behavioral therapy; CCUs, combustible cigarette users; ECUs, e-cigarette users.

##### 4.3.3.4 Adult ECU cessation: harm-reduction framing and perceived control

For adult ECUs seeking cessation, intervention framing may be most acceptable when it acknowledges autonomy and harm-reduction goals, while still specifying a clear pathway toward nicotine discontinuation (19, 44). Where clinically appropriate, integrating pharmacotherapy (e.g., NRT) with digitally delivered behavioral support can be considered, with feasibility evidence suggesting that this combination is implementable for adult ECUs (34). Because e-cigarette-specific “perceived control” and convenience-driven use can sustain frequent nicotine intake, adult ECU interventions should explicitly address these barriers through stepwise goal-setting and structured self-monitoring (24, 44).

### 4.4 Policy implementation

Regarding evidence base, this section integrates evidence from nine sources (systematic review/meta-analysis, *n* = 2; RCT, *n* = 1; cross-sectional, *n* = 2; qualitative, *n* = 2; quasi-experimental, *n* = 1; narrative review, *n* = 1) to derive implementation-relevant implications for differentiated cessation pathways, prevention priorities, regulation/quality assurance, and monitoring systems for CCUs and ECUs (21, 23, 29, 32, 34, 35, 41, 44, 89).

#### 4.4.1 Integration within healthcare systems

The evidence synthesized here indicates that CCUs and ECUs differ in dependence mechanisms and responsiveness to intervention modality/intensity. CCUs show the most consistent benefit from structured, high-intensity, multicomponent interventions integrating CBT, MI, and pharmacotherapy (32, 41, 46), while ECUs, particularly adolescents and young adults, demonstrate greater engagement with digitally delivered, lower-intensity, and socially embedded interventions (34, 44). These patterns support the consideration of a differentiated stepped-care pathway. For CCUs, integration of structured behavioral treatment within primary care and specialized services aligns with guideline recommendations (46). For ECUs, digitally delivered and peer-oriented interventions may serve as frontline or adjunctive approaches, with automated re-engagement features addressing attrition (35).

#### 4.4.2 Education and developmentally informed prevention

The literature highlights the influence of peer norms, sensory appeal, and attenuated risk perception in adolescent ECU use (21, 23, 29). MI-informed and text-based interventions improve motivation and short- to mid-term cessation outcomes among youth (44). Thus, prevention may be more effective in this group when addressing identity formation and social reinforcement rather than relying solely on long-term risk messaging (14). Accordingly, prevention strategies incorporating peer-norm modification, self-determination skills, and media literacy components may enhance engagement and effectiveness in younger populations (44).

#### 4.4.3 Regulation, accessibility, and quality assurance

The rapid expansion of digital cessation platforms, particularly for ECUs, has been accompanied by heterogeneity in theoretical grounding and supporting evidence (34, 85). Although digital interventions show promising engagement and short-term outcomes, standardized evaluation frameworks remain limited (35). These observations support the potential value of transparent evaluation and certification systems for digital cessation tools (85). Age-sensitive access regulation may also be justified given the developmental vulnerability and sensory–social reinforcement mechanisms observed in adolescent ECUs (21).

#### 4.4.4 Monitoring and evaluation to support implementation

The review underscores the limited availability of longitudinal comparative data and the need for standardized outcome metrics (32). Digital tools, including app-based tracking and wearable-linked systems, enable the real-time monitoring of use patterns and support adaptive, mechanism-informed intervention delivery (34, 89). The linkage of longitudinal cohort data with standardized cessation outcomes may facilitate long-term effect evaluation and support ongoing refinement of differentiated cessation models.

### 4.5 Future research directions

Several critical gaps must be addressed to advance the field of tobacco cessation research. First, longitudinal studies directly comparing intervention effectiveness between CCUs and ECUs using standardized outcome measures are required to establish evidence-based treatment algorithms. Second, investigations into dual users, who represent a significant and growing population, are crucial as their cessation needs may differ from those of single-product users. Third, the rapid evolution of digital health technologies presents opportunities for precision medicine approaches to tobacco cessation, such as through AI-driven adaptive interventions that can personalize content, timing, and intensity based on real-time behavioral data and user characteristics. Additionally, examinations into the long-term effectiveness and cost-effectiveness of digital interventions compared to traditional approaches are crucial for enabling well-informed healthcare policy decisions. Fourth, future research should clarify the role of pharmacotherapy across product–user subgroups. Pharmacotherapy (e.g., NRT, varenicline, and bupropion) remains an evidence-based cornerstone for CCUs when combined with behavioral support, whereas evidence for traditional pharmacotherapies among ECUs—particularly adolescents and young adults—remains scant, with no agents currently approved specifically for e-cigarette cessation. Further examinations should determine whether nicotine tapering or novel pharmacologic approaches (e.g., nicotine receptor modulators) can support e-cigarette cessation without compromising abstinence verification or outcome measurement. Future studies should also examine optimal integration models for pharmacological and non-pharmacological interventions among dual users and ECUs with high nicotine dependence.

Building on this differentiated behavioral conceptual framework, future studies should test whether matching dominant behavioral mechanisms (e.g., withdrawal-driven vs. sensory/social reinforcement–driven use) to intervention modality and intensity yields superior cessation outcomes across CCUs, ECUs, and dual users. Comparative and longitudinal designs should be conducted to empirically validate the mechanism-informed personalization of non-pharmacological combustible cigarette cessation and e-cigarette cessation interventions.

### 4.6 Strengths and limitations

#### 4.6.1 Strengths

This review has several strengths and unique contributions. First, it establishes a comparative framework by systematically analyzing CCUs and ECUs from a single perspective, moving beyond effect comparisons to examine psychological mechanisms and dependence patterns. Second, it integrates diverse evidence from CBT, MI, digital health, and quitline studies. Third, this review provides clinically actionable recommendations for healthcare settings, addresses a key gap by focusing on emerging ECU populations (e.g., adolescents and young adults), and enhances policy relevance by linking evidence to public health strategies. Finally, it upholds methodological transparency by acknowledging the limitations of the narrative approach and outlining future research directions.

#### 4.6.2 Limitations

This review has several limitations that warrant cautious interpretations of the findings. As a narrative review, it did not employ a fully systematic search strategy or meta-analytic methods consistent with PRISMA guidelines (95); therefore, selection bias cannot be excluded. The differentiated behavioral conceptual framework was inductively derived from the narrative synthesis rather than quantitatively validated or experimentally tested and should be regarded as hypothesis-generating rather than a definitive causal model. Moreover, overlap in nicotine reinforcement processes across CCUs and ECUs suggests that the distinctions proposed reflect differences in relative salience rather than mutually exclusive mechanisms. The evidence base for ECU cessation interventions also remains limited and heterogeneous across age groups, device types, and usage patterns. While many digital interventions report short-term outcomes, long-term comparative data are scarce. Rapid technological evolution in both e-cigarettes and digital health platforms may further limit the temporal stability of the current findings. Studies also varied in how they defined and categorized user groups (e.g., exclusive CCUs, exclusive ECUs, dual users, or mixed/unclear samples), and exclusive versus dual use was not consistently distinguished across datasets. This heterogeneity limits the ability to draw firm user-type-specific conclusions and constrains generalizability across CCUs, ECUs, and dual users. Future research should adopt standardized and transparent user-group definitions (e.g., clear time windows and exclusivity criteria) and report outcomes separately by user type. Finally, heterogeneity in outcome measures, follow-up duration, and intervention design constrains direct cross-product comparisons. Future research should prioritize standardized outcomes, longer follow-up, and comparative designs that explicitly distinguish exclusive CCUs, exclusive ECUs, and dual users.

## 5 Conclusion

This review synthesizes the effectiveness of non-pharmacological smoking cessation interventions while considering the psychological, behavioral, and contextual characteristics of CCUs and ECUs. For CCUs, pronounced physiological withdrawal and automated smoking habits make high-intensity, structured interventions most appropriate, with CBT as the core approach supported by MI and pharmacotherapy. For ECUs, intervention acceptability is affected by social and sensory reinforcement and a lower risk perception, indicating greater effectiveness for stepwise goals (e.g., reducing frequency, lowering nicotine concentration, progressing to nicotine-free use, and ultimately cessation) combined with digital and peer-based approaches. Age-specific tailoring is also crucial; digital engagement and peer/identity-based strategies may be particularly effective for adolescent and young ECUs, whereas older CCUs may benefit more from face-to-face or telephone support and clear health risk messaging. Overall, effective tobacco control requires differentiated strategies that reflect the distinct characteristics of products and users. Collaboration among healthcare systems, policymakers, and researchers is essential to develop and implement these tailored strategies while strengthening the evidence base for optimal intervention design and delivery.

## Supplementary material

SUPPLEMENTARY TABLE S1 Full search terms and results for each database.

SUPPLEMENTARY TABLE S2 Inclusion and exclusion criteria.

SUPPLEMENTARY TABLE S3 Overview of included studies with characteristics, populations, interventions, key findings, and thematic mapping.

## References

[1] World Health Organization. (2025). WHO report on the global tobacco epidemic, 2025: warning about the dangers of tobacco. Available online at: https://www.who.int/publications/i/item/9789240112063 (Accessed January 2, 2026)

[2] Carter P Lagan J Fortune C Bhatt DL Vestbo J Niven R . Association of cardiovascular disease with respiratory disease. J Am Coll Cardiol. (2019) 73:2166–77. doi: 10.1016/j.jacc.2018.11.063, 30846341

[3] Roth GA Mensah GA Johnson CO Addolorato G Ammirati E Baddour LM . Global burden of cardiovascular diseases and risk factors, 1990–2019: update from the GBD 2019 study. J Am Coll Cardiol. (2020) 76:2982–3021. doi: 10.1016/j.jacc.2020.11.010, 33309175 PMC7755038

[4] Goodchild M Nargis N Tursan d’Espaignet ET. Global economic cost of smoking-attributable diseases. Tob Control. (2018) 27:58–64. doi: 10.1136/tobaccocontrol-2016-053305, 28138063 PMC5801657

[5] GBD 2019 Tobacco Collaborators. Spatial, temporal, and demographic patterns in prevalence of smoking tobacco use and attributable disease burden in 204 countries and territories, 1990–2019: a systematic analysis from the global burden of disease study 2019. Lancet. (2021) 397:2337–60. doi: 10.1016/S0140-6736(21)01169-7, 34051883 PMC8223261

[6] Korea Disease Control and Prevention Agency. (2024). The 20th Korea youth health behavior survey statistics (2024). Available online at: https://kdca.go.kr/yhs/yhs/main.do (Accessed January 2, 2026)

[7] Park SH Duncan DT El Shahawy OE Lee L Shearston JA Tamura K . Characteristics of adults who switched from cigarette smoking to e-cigarettes. Am J Prev Med. (2017) 53:652–60. doi: 10.1016/j.amepre.2017.06.033, 28864130 PMC5983046

[8] Erhabor J Boakye E Obisesan O Osei AD Tasdighi E Mirbolouk H . E-cigarette use among US adults in the 2021 behavioral risk factor surveillance system survey. JAMA Netw Open. (2023) 6:e2340859. doi: 10.1001/jamanetworkopen.2023.40859, 37921768 PMC10625038

[9] Wang Y Sung H-Y Lea Watkins SL Lightwood J Yao T Max W. The association of current exclusive e-cigarette use and dual use of e-cigarettes and cigarettes with psychological distress among U.S. adults. Prev Med Rep. (2023) 36:102425. doi: 10.1016/j.pmedr.2023.102425, 37810268 PMC10556823

[10] Shulla K. Leal-Filho W. (2023). Achieving the UN agenda 2030: overall actions for the successful implementation of the sustainable development goals before and after the 2030 deadline. European Union Parliament Available online at: https://www.europarl.europa.eu/thinktank/en/document/EXPO_IDA(2022)702576 (Accessed January 2, 2026)

[11] Tindle HA Stevenson Duncan M Greevy RA Vasan RS Kundu S Massion PP . Lifetime smoking history and risk of lung cancer: results from the Framingham heart study. J Natl Cancer Inst. (2018) 110:1201–7. doi: 10.1093/jnci/djy041, 29788259 PMC6235683

[12] Thomson B Islami F. Association of smoking cessation and cardiovascular, cancer, and respiratory mortality. JAMA Intern Med. (2024) 184:110–2. doi: 10.1001/jamainternmed.2023.6419, 38010645 PMC10682940

[13] Blashki GA Piterman L Meadows GN Clarke DM Prabaharan V Gunn JM . Impact of an educational intervention on general practitioners’ skills in cognitive behavioural strategies: a randomised controlled trial. Med J Aust. (2008) 188:S129–32. doi: 10.5694/j.1326-5377.2008.tb01876.x 18558913

[14] Colby SM Nargiso J Tevyaw TOL Barnett NP Metrik J Lewander W . Enhanced motivational interviewing versus brief advice for adolescent smoking cessation: results from a randomized clinical trial. Addict Behav. (2012) 37:817–23. doi: 10.1016/j.addbeh.2012.03.011 22472523 PMC3356495

[15] Martínez-Vispo C Rodríguez-Cano R López-Durán A Senra C Fernández del Río E Becoña E. Cognitive-behavioral treatment with behavioral activation for smoking cessation: randomized controlled trial. PLoS One. (2019) 14:e0214252. doi: 10.1371/journal.pone.0214252, 30958831 PMC6453447

[16] Alshehri FS. An overview of traditional smoking cessation interventions and E-cigarettes. Front Pharmacol. (2024) 15:1293062. doi: 10.3389/fphar.2024.1293062, 39104396 PMC11298375

[17] World Health Organization. (2021). WHO report on the global tobacco epidemic 2021: addressing new and emerging products. Available online at: https://www.who.int/publications/i/item/9789240032095 (Accessed January 2, 2026)

[18] Green BN Johnson CD Adams A. Writing narrative literature reviews for peer-reviewed journals: secrets of the trade. J Chiropr Med. (2006) 5:101–17. doi: 10.1016/s0899-3467(07)60142-6, 19674681 PMC2647067

[19] Kim YA Lee J Kim J Kim J Ko Y-J Kim S. The characteristics of electronic cigarette user among Korean smokers: the sixth Korean national health and nutrition examination survey, 2013–2015. Korean J Fam Pract. (2019) 9:324–30. doi: 10.21215/kjfp.2019.9.4.324

[20] Meza R Cao P Jeon J Warner KE Levy DT. Trends in US adult smoking prevalence, 2011 to 2022. JAMA Health Forum. (2023) 4:e234213. doi: 10.1001/jamahealthforum.2023.4213, 38038988 PMC10692849

[21] Cooper M Harrell MB Pérez A Delk J Perry CL. Flavorings and perceived harm and addictiveness of e-cigarettes among youth. Tob Regul Sci. (2016) 2:278–89. doi: 10.18001/TRS.2.3.7, 27722185 PMC5049876

[22] Gaiha SM Lempert LK McKelvey K Halpern-Felsher B. E-cigarette devices, brands, and flavors attract youth: informing FDA’S policies and priorities to close critical gaps. Addict Behav. (2022) 126:107179. doi: 10.1016/j.addbeh.2021.107179, 34861522 PMC8712419

[23] Groom AL Vu TT Landry RL Kesh A Hart JL Walker KL . The influence of friends on teen vaping: a mixed-methods approach. Int J Environ Res Public Health. (2021) 18:6784. doi: 10.3390/ijerph18136784, 34202600 PMC8296881

[24] Rhoades DA Comiford AL Dvorak JD Ding K Hopkins M Spicer P . Vaping patterns, nicotine dependence and reasons for vaping among American Indian dual users of cigarettes and electronic cigarettes. BMC Public Health. (2019) 19:1211. doi: 10.1186/s12889-019-7523-5, 31477072 PMC6721166

[25] Etter JF Bullen C. Electronic cigarette: users profile, utilization, satisfaction and perceived efficacy. Addiction. (2011) 106:2017–28. doi: 10.1111/j.1360-0443.2011.03505.x, 21592253

[26] Harlow AF Cho J Tackett AP McConnell RS Leventhal AM Stokes AC . Motivations for E-cigarette use and associations with vaping frequency and smoking abstinence among adults who smoke cigarettes in the United States. Drug Alcohol Depend. (2022) 238:109583. doi: 10.1016/j.drugalcdep.2022.109583, 35907310 PMC9994580

[27] Benowitz NL. Nicotine addiction. N Engl J Med. (2010) 362:2295–303. doi: 10.1056/NEJMra0809890, 20554984 PMC2928221

[28] Morean ME Krishnan-Sarin S Sussman S Foulds J Fishbein H Grana R . Development and psychometric validation of a novel measure of sensory expectancies associated with E-cigarette use. Addict Behav. (2019) 91:208–15. doi: 10.1016/j.addbeh.2018.08.031 30197032 PMC6358482

[29] Pokhrel P Herzog TA Muranaka N Fagan P. Young adult e-cigarette users’ reasons for liking and not liking e-cigarettes: a qualitative study. Psychol Health. (2015) 30:1450–69. doi: 10.1080/08870446.2015.1061129, 26074148 PMC4657726

[30] Pokhrel P Lam TH Pagano I Kawamoto CT Herzog TA. Young adult e-cigarette use outcome expectancies: validity of a revised scale and a short scale. Addict Behav. (2018) 78:193–9. doi: 10.1016/j.addbeh.2017.11.019, 29195147 PMC5783754

[31] DiClemente CC Prochaska JO Fairhurst SK Velicer WF Velasquez MM Rossi JS. The process of smoking cessation: an analysis of precontemplation, contemplation, and preparation stages of change. J Consult Clin Psychol. (1991) 59:295–304. doi: 10.1037//0022-006x.59.2.295 2030191

[32] Hartmann-Boyce J Livingstone-Banks J Ordóñez-Mena JM Fanshawe TR Lindson N Freeman SC . Behavioural interventions for smoking cessation: an overview and network meta-analysis. Cochrane Database Syst Rev. (2021) 2021:CD013229. doi: 10.1002/14651858.CD013229.pub2, 33411338 PMC11354481

[33] Vinci C. Cognitive behavioral and mindfulness-based interventions for smoking cessation: a review of the recent literature. Curr Oncol Rep. (2020) 22:58. doi: 10.1007/s11912-020-00915-w, 32415381 PMC7874528

[34] Webb J Lin Y-T Ang A Michero D Majeed A Eisingerich A . Feasibility and preliminary outcomes of a mobile intervention combining cognitive behavioral therapy, virtual coaching, and nicotine replacement therapy for nicotine vaping cessation. Telemed Rep. (2023) 4:48–52. doi: 10.1089/tmr.2023.0009, 37102136 PMC10125401

[35] Whittaker R McRobbie H Bullen C Rodgers A Gu Y Dobson R. Mobile phone text messaging and app-based interventions for smoking cessation. Cochrane Database Syst Rev. (2019) 10:CD006611. doi: 10.1002/14651858.CD006611.pub5, 31638271 PMC6804292

[36] Beck JS. Cognitive Behavior Therapy: Basics and beyond. New York: The Guilford Press (2011).

[37] Bandura A. Social Foundations of Thought and Action. Englewood Cliffs, NJ: Prentice-Hall, Inc. (1986).

[38] Prochaska JO DiClemente CC. Stages and processes of self-change of smoking: toward an integrative model of change. J Consult Clin Psychol. (1983) 51:390–5. doi: 10.1037//0022-006x.51.3.390, 6863699

[39] Ryan RM Deci EL. Intrinsic and extrinsic motivations: classic definitions and new directions. Contemp Educ Psychol. (2000) 25:54–67. doi: 10.1006/ceps.1999.1020, 10620381

[40] Carroll KM. Lost in translation? Moving contingency management and cognitive behavioral therapy into clinical practice. Ann N Y Acad Sci. (2014) 1327:94–111. doi: 10.1111/nyas.12501, 25204847 PMC4206586

[41] Celik ZH Sevi OM. Effectiveness of cognitive behavioral therapy for smoking cessation: a systematic review. Curr Approaches Psychiatry. (2020) 12:54–71. doi: 10.18863/pgy.534638

[42] Lancaster T Stead LF. Individual behavioural counselling for smoking cessation. Cochrane Database Syst Rev. (2017) 2018:CD001292. doi: 10.1002/14651858.CD001292.pub3, 12137623

[43] Rüther T Kiss A Eberhardt K Linhardt A Kröger C Pogarell O. Evaluation of the cognitive behavioral smoking reduction program “Smoke_less”: a randomized controlled trial. Eur Arch Psychiatry Clin Neurosci. (2018) 268:269–77. doi: 10.1007/s00406-017-0818-6, 28616772

[44] Sanchez S Kaufman P Pelletier H Baskerville B Feng P O’Connor S . Is vaping cessation like smoking cessation? A qualitative study exploring the responses of youth and young adults who vape e-cigarettes. Addict Behav. (2021) 113:106687. doi: 10.1016/j.addbeh.2020.106687, 33045643

[45] Stead LF Carroll AJ Lancaster T. Group behaviour therapy programmes for smoking cessation. Cochrane Database Syst Rev. (2017) 2017:CD001007. doi: 10.1002/14651858.CD001007.pub3, 28361497 PMC6464070

[46] Fiore M Jaén CR Baker TB Bailey WC Benowitz NL Curry SJ . Treating Tobacco Use and Dependence: 2008 Update. Rockville, MD: U.S. Department of Health and Human Services (2009).

[47] Perkins KA. Cognitive-Behavioral Therapy for Smoking Cessation: a Practical Guidebook to the Most Effective Strategies. New York: Taylor & Francis (2025).

[48] Bandura A. Self-Efficacy: the Exercise of Control. New York: W. H. Freeman and Company (1997).

[49] Shiffman S. Paty J. A. Kassel J. D. Gnys M. Zettler-Segal M. (2005). “Cognitive-behavioral therapy for smoking cessation: a practical guidebook to the most effective strategies,” in Coping with Tobacco Dependence: a Guide for Patients and Clinicians, eds. Stitzer J. D. Wit M. W. de (Cambridge, UK: Cambridge University Press), 115–139.

[50] Bendotti H Marshall HM Gartner C Ireland D Lawler S. Identifying motivational interviewing techniques in Quitline smoking cessation counselling sessions from Queensland, Australia. J Health Psychol. (2025) 30:1653–64. doi: 10.1177/13591053241274091, 39219274 PMC12166157

[51] Caponnetto P DiPiazza J Cappello GC Demma S Maglia M Polosa R. Multimodal smoking cessation in a real-life setting: combining motivational interviewing with official therapy and reduced risk products. Tob Use Insights. (2019) 12:1179173X19878435. doi: 10.1177/1179173X19878435, 31636483 PMC6783661

[52] Gill VS Chaudhary N Randhawa A Verma M Rai GK Mishra S. A prospective study to assess the outcome of motivational interviewing among male students of Haryana, India: a strive towards smoking cessation in the youth. Cureus. (2022) 14:e22642. doi: 10.7759/cureus.22642, 35371670 PMC8964476

[53] Hettema J Steele J Miller WR. Motivational interviewing. Annu Rev Clin Psychol. (2005) 1:91–111. doi: 10.1146/annurev.clinpsy.1.102803.143833, 17716083

[54] Lindson-Hawley N Thompson TP Begh R. Motivational interviewing for smoking cessation. Cochrane Database Syst Rev. (2015) 3:CD006936. doi: 10.1002/14651858.CD006936.pub3 25726920

[55] Miller WR Rollnick S. Motivational Interviewing: Helping People Change. New York: The Guilford Press (2012).

[56] Sikka G Oluyinka M Schreiber R Galiatsatos P. Electronic cigarette cessation in youth and young adults: a case series. Tob Use Insights. (2021) 14:1179173X211026676. doi: 10.1177/1179173X211026676 PMC8216413 34211303

[57] Santa Ana EJ LaRowe SD Gebregziabher M Morgan-Lopez AA Lamb K Beavis KA . Randomized controlled trial of group motivational interviewing for veterans with substance use disorders. Drug Alcohol Depend. (2021) 223:108716. doi: 10.1016/j.drugalcdep.2021.108716, 33873028 PMC9011162

[58] Centis E Petroni ML Ghirelli V Cioni M Navacchia P Guberti E . Motivational interviewing adapted to group setting for the treatment of relapse in the behavioral therapy of obesity. A clinical audit. Nutrients. (2020) 12:3881. doi: 10.3390/nu12123881, 33353057 PMC7765885

[59] Abroms LC Boal AL Simmens SJ Mendel JA Windsor RA. A randomized trial of Text2Quit: a text messaging program for smoking cessation. Am J Prev Med. (2014) 47:242–50. doi: 10.1016/j.amepre.2014.04.010, 24913220 PMC4545234

[60] Byaruhanga J Paul CL Wiggers J Byrnes E Mitchell A Lecathelinais C . Connectivity of real-time video counselling versus telephone counselling for smoking cessation in rural and remote areas: an exploratory study. Int J Environ Res Public Health. (2020) 17:2891. doi: 10.3390/ijerph17082891, 32331356 PMC7215336

[61] Byaruhanga J Wiggers J Paul CL Byrnes E Mitchell A Lecathelinais C . Acceptability of real-time video counselling compared to other behavioural interventions for smoking cessation in rural and remote areas. Drug Alcohol Depend. (2020) 217:108296. doi: 10.1016/j.drugalcdep.2020.108296, 32980788 PMC7491422

[62] Cartujano-Barrera F Sanderson Cox L Arana-Chicas E Ramírez M Perales-Puchalt J Valera P . Feasibility and acceptability of a culturally- and linguistically-adapted smoking cessation text messaging intervention for Latino smokers. Front Public Health. (2020) 8:269. doi: 10.3389/fpubh.2020.00269, 32714891 PMC7344180

[63] Free C Knight R Robertson S Whittaker R Edwards P Zhou W . Smoking cessation support delivered via mobile phone text messaging (txt2stop): a single-blind, randomised trial. Lancet. (2011) 378:49–55. doi: 10.1016/S0140-6736(11)60701-0, 21722952 PMC3143315

[64] Graham AL Amato MS Cha S Jacobs MA Bottcher MM Papandonatos GD. Effectiveness of a vaping cessation text message program among young adult e-cigarette users: a randomized clinical trial. JAMA Intern Med. (2021) 181:923–30. doi: 10.1001/jamainternmed.2021.1793, 33999133 PMC8129897

[65] Graham AL Cha S Jacobs MA Amato MS Funsten AL Edwards G . A vaping cessation text message program for adolescent e-cigarette users: a randomized clinical trial. JAMA. (2024) 332:713–21. doi: 10.1001/jama.2024.11057, 39110436 PMC11307165

[66] Hartmann-Boyce J. Telephone counselling for smoking cessation. Cochrane Datab Syst Rev. (2019) 2019:CD002850. doi: 10.1002/14651858.CD002850.pub4, 31045250 PMC6496404

[67] Skov-Ettrup LS Dalum P Bech M Tolstrup JS. The effectiveness of telephone counselling and internet- and text-message-based support for smoking cessation: results from a randomized controlled trial. Addiction. (2016) 111:1257–66. doi: 10.1111/add.13302, 26748541

[68] Stead LF Lancaster T. Combined pharmacotherapy and behavioural interventions for smoking cessation. Cochrane Database Syst Rev. (2012) 10:CD008286. doi: 10.1002/14651858.CD008286.pub2 23076944

[69] Tzelepis F Paul CL Williams CM Gilligan C Regan T Daly J . Real-time video counselling for smoking cessation. Cochrane Database Syst Rev. (2019) 2019:CD012659. doi: 10.1002/14651858.CD012659.pub2, 31684699 PMC6818086

[70] Chen S Tang J Wu C Zhang G Zhang J Liao Y. Preliminary efficacy of a cognitive behavioral therapy–based smartphone app for smoking cessation in China: randomized controlled pilot trial. JMIR Form Res. (2024) 8:e48050. doi: 10.2196/48050, 38498030 PMC10985609

[71] Etter J-F Vera Cruz G Khazaal Y. Predicting smoking cessation, reduction and relapse six months after using the stop-Tabac app for smartphones: a machine learning analysis. BMC Public Health. (2023) 23:1076. doi: 10.1186/s12889-023-15859-6, 37277740 PMC10242904

[72] Heffner JL Baker K Georgiou K Graham AL Kelly MM Konstantinou P . ACT on vaping: pilot randomized controlled trial of a novel digital health app with text messaging for young adult vaping cessation. Nicotine Tob Res. (2025) 28:720–30. doi: 10.1093/ntr/ntaf112, 40411791 PMC13101998

[73] Imran Ho DSHI Jabir F Sallahuddin SN Ahwan NAM Sathiyaseelan G Zahari MI . The impact of gamification on smoking cessation: a systematic review and meta-analysis. Tob Induc Dis. (2025) 23:1–18. doi: 10.18332/tid/203937, 40575736 PMC12199787

[74] Lung HH Tan XY Rosencovich N Goldenhersch E Waitman C Halpern-Felsher B. 143. Acceptability of “back to school”: a novel minecraft-based E-cigarette prevention and cessation game. J Adolesc Health. (2024) 74:S76–7. doi: 10.1016/j.jadohealth.2023.11.342

[75] Marler JD Fujii CA Galanko JA Balbierz DJ Utley DS. Durability of abstinence after completing a comprehensive digital smoking cessation program incorporating a mobile app, breath sensor, and coaching: cohort study. J Med Internet Res. (2021) 23:e25578. doi: 10.2196/25578, 33482628 PMC7920755

[76] Marler JD Fujii CA Utley MT Balbierz DJ Galanko JA Utley DS. Outcomes of a comprehensive mobile vaping cessation program in adults who vape daily: cohort study. JMIR Form Res. (2024) 8:e57376. doi: 10.2196/57376, 39331522 PMC11555445

[77] Martinez Agulleiro LM Patil B Firth J Sawyer C Amann BL Fonseca F . A systematic review of digital interventions for smoking cessation in patients with serious mental illness. Psychol Med. (2023) 53:4856–68. doi: 10.1017/S003329172300123X, 37161690 PMC10476065

[78] Masaki K Tateno H Nomura A Muto T Suzuki S Satake K . A randomized controlled trial of a smoking cessation smartphone application with a carbon monoxide checker. NPJ Digit Med. (2020) 3:35. doi: 10.1038/s41746-020-0243-5, 32195370 PMC7067789

[79] Perski O Crane D Beard E Brown J. Does the addition of a supportive chatbot promote user engagement with a smoking cessation app? An experimental study. Digit Health. (2019) 5:2055207619880676. doi: 10.1177/2055207619880676, 31620306 PMC6775545

[80] Rajani NB Bustamante L Weth D Romo L Mastellos N Filippidis FT. Engagement with gamification elements in a smoking cessation app and short-term smoking abstinence: quantitative assessment. JMIR Serious Games. (2023) 11:e39975. doi: 10.2196/39975, 36724003 PMC9932870

[81] Shi J Fu R Hamilton H Chaiton M. A machine learning approach to predict e-cigarette use and dependence among Ontario youth. Health Promot Chronic Dis Prev Can. (2022) 42:21–8. doi: 10.24095/hpcdp.42.1.04, 35044141 PMC9067014

[82] Webb J Peerbux S Ang A Siddiqui S Sherwani Y Ahmed M . Long-term effectiveness of a clinician-assisted digital cognitive behavioral therapy intervention for smoking cessation: secondary outcomes from a randomized controlled trial. Nicotine Tob Res. (2022) 24:1763–72. doi: 10.1093/ntr/ntac113, 35470860 PMC9597001

[83] Webb J Peerbux S Smittenaar P Siddiqui S Sherwani Y Ahmed M . Preliminary outcomes of a digital therapeutic intervention for smoking cessation in adult smokers: randomized controlled trial. JMIR Ment Health. (2020) 7:e22833. doi: 10.2196/22833, 33021488 PMC7576529

[84] Weser VU Duncan LR Sands BE Schartmann A Jacobo S François B . Evaluation of a virtual reality E-cigarette prevention game for adolescents. Addict Behav. (2021) 122:107027. doi: 10.1016/j.addbeh.2021.107027, 34225030

[85] Liao Y Tang J. Efficacy of cognitive behavioural therapy-based smartphone app for smoking cessation in China: a study protocol of a randomised controlled trial. BMJ Open. (2021) 11:e041985. doi: 10.1136/bmjopen-2020-041985, 33441359 PMC7812100

[86] Hartmann-Boyce J Hong B Livingstone-Banks J Wheat H Fanshawe TR. Additional behavioural support as an adjunct to pharmacotherapy for smoking cessation. Cochrane Database Syst Rev. (2019) 2019:CD009670. doi: 10.1002/14651858.CD009670.pub4, 31166007 PMC6549450

[87] Puljević C Meciar I Holland A Stjepanović D Snoswell CL Thomas EE . Systematic review and meta-analysis of text messaging interventions to support tobacco cessation. Tob Control. (2025) 34:228–38. doi: 10.1136/tc-2023-058323, 38448226

[88] Skov-Ettrup LS Dalum P Ekholm O Tolstrup JS. Reach and uptake of internet- and phone-based smoking cessation interventions: results from a randomized controlled trial. Prev Med. (2014) 62:38–43. doi: 10.1016/j.ypmed.2014.01.020, 24508983

[89] Palmer AM Tomko RL Squeglia LM Gray KM Carpenter MJ Smith TT . A pilot feasibility study of a behavioral intervention for nicotine vaping cessation among young adults delivered via telehealth. Drug Alcohol Depend. (2022) 232:109311. doi: 10.1016/j.drugalcdep.2022.109311, 35123362 PMC8885867

[90] Klemperer EM Villanti AC. Why and how do dual users quit vaping? Survey findings from adults who use electronic and combustible cigarettes. Tob Induc Dis. (2021) 19:12. doi: 10.18332/tid/132547, 33603595 PMC7885258

[91] Martinez U Simmons VN Sutton SK Drobes DJ Meltzer LR Brandon KO . Targeted smoking cessation for dual users of combustible and electronic cigarettes: a randomised controlled trial. Lancet Public Health. (2021) 6:e500–9. doi: 10.1016/S2468-2667(20)30307-8, 34175001 PMC8281505

[92] Meltzer LR Simmons VN Piñeiro B Drobes DJ Quinn GP Meade CD . Development of a self-help smoking cessation intervention for dual users of tobacco cigarettes and e-cigarettes. Int J Environ Res Public Health. (2021) 18:2328. doi: 10.3390/ijerph18052328, 33673413 PMC7956571

[93] Vickerman KA Carpenter KM Miles LN Hsu JM Watt KA Brandon TH . Treatment development, implementation, and participant baseline characteristics: a randomized pilot study of a tailored quitline intervention for individuals who smoke and vape. Contemp Clin Trials Commun. (2021) 24:100845. doi: 10.1016/j.conctc.2021.100845, 34568637 PMC8449159

[94] Killen JD Fortmann SP Schatzberg AF Arredondo C Murphy G Hayward C . Extended cognitive behavior therapy for cigarette smoking cessation. Addiction. (2008) 103:1381–90. doi: 10.1111/j.1360-0443.2008.02273.x, 18855829 PMC4119230

[95] Page MJ McKenzie JE Bossuyt PM Boutron I Hoffmann TC Mulrow CD . The PRISMA 2020 statement: an updated guideline for reporting systematic reviews. BMJ. (2021) 372:n71. doi: 10.1136/bmj.n71, 33782057 PMC8005924

[96] World Health Organization. (2019). WHO report on the global tobacco epidemic 2019: offer help to quit tobacco use. Available online at: https://www.who.int/publications-detail-redirect/9789241516204 (Accessed January 2, 2026)

[97] Moher D Liberati A Tetzlaff J Altman DG. Preferred reporting items for systematic reviews and meta-analyses: the PRISMA statement. BMJ. (2009) 339:b2535. doi: 10.1136/bmj.b2535, 19622551 PMC2714657

[98] Vahratian A Briones EM Jamal A Marynak KL. Electronic cigarette use among adults in the United States, 2019–2023. NCHS Data Brief, no. 524. Hyattsville, MD: National Center for Health Statistics. (2025) doi: 10.15620/cdc/174583, 40036117 PMC12035660

[99] Graham AL Jacobs MA Amato MS Cha S Bottcher MM Papandonatos GD. Effectiveness of a quit vaping text message program in promoting abstinence among young adult e-cigarette users: protocol for a randomized controlled trial. JMIR Res. Protoc. (2020) 9:e18327. doi: 10.2196/18327, 32356774 PMC7229526

