# Supplementary material for: Psychological and behavioral profiles of combustible vs. e-cigarette users: a narrative review of the implications for tailored non-pharmacological cessation interventions
Source: Front Public Health. 2026 Jun 17;14:1779824. doi: 10.3389/fpubh.2026.1779824 (PMC13318882; doi:10.3389/fpubh.2026.1779824)
Supplement: SUPPLEMENTARY TABLE S1 — Full search terms and results for each database. [file Table_1.DOCX]

Supplementary Material

# Table S1. Full search terms and results for each database

| Databases: PubMed (Date of search: 11.13.2025) | | | | | | | | |
| --- | --- | --- | --- | --- | --- | --- | --- | --- |
| Component | **Key word** | | | **#** | | **Search terms** | **Filters** | Results |
| Population | Combustible cigarette | | | 1 | | "combustible cigarette"[Title/Abstract] | | 467 |
|  | E-cigarette / vaping / dual use | | | 2 | | ("e-cigarette"[Title/Abstract] OR vaping[Title/Abstract] OR "dual use"[Title/Abstract]) | | 11,797 |
|  | Population combined | | | 3 | | #1 OR #2 (with date filter: 2010/01/01:2025/11/13[pdat]) | | 11,942 |
| Intervention | Cessation interventions | | | 4 | | "smoking cessation"[Title/Abstract] OR "cognitive behavioral therapy"[Title/Abstract] OR "motivational interviewing"[Title/Abstract] OR "digital health"[Title/Abstract] OR "quitline"[Title/Abstract] OR "text messaging"[Title/Abstract] AND (2010/01/01:2025/11/13[pdat]) | | 62,507 |
| P & I | Population AND intervention | | | 5 | | #3 AND #4 (with date filter: 2010/01/01:2025/11/13[pdat]) | | 1,507 |
| Study Design | English | | | 6 | | #5 AND english[Language] | | 1,464 |
|  | English + Humans | | | 7 | | #6 AND humans[MeSH Terms] | | 1,113 |
| Databases: Scopus (Date of search: 11.13.2025) | | | | | | | | |
| Component | | **Key word** | | **#** | **Search terms** | | | Results |
| Population | | Tobacco product user  group terms | | 1 | TITLE-ABS ( "combustible cigarette" OR "e-cigarette" OR "vaping" OR "dual use" ) AND PUBYEAR > 2009 AND PUBYEAR < 2026 | | | 17,226 |
| Intervention | | Non-pharmacological cessation intervention  terms | | 2 | TITLE-ABS ( "smoking cessation" OR "cognitive behavioral therapy" OR "motivational interviewing" OR "digital health" OR "quitline" OR "text messaging" ) AND PUBYEAR > 2009 AND PUBYEAR < 2026 | | | 76,754 |
| P & I | | Population AND  Intervention | | 3 | TITLE-ABS ( ( "combustible cigarette" OR "e-cigarette" OR "vaping" OR "dual use" ) AND ( "smoking cessation" OR "cognitive behavioral therapy" OR "motivational interviewing" OR "digital health" OR "quitline" OR "text messaging" ) ) AND PUBYEAR > 2009 AND PUBYEAR < 2026 | | | 1,844 |
| P & I + limits | | Final (Identification count for PRISMA) | | 4 | TITLE-ABS ( ( "combustible cigarette" OR "e-cigarette" OR "vaping" OR "dual use" ) AND ( "smoking cessation" OR "cognitive behavioral therapy" OR "motivational interviewing" OR "digital health" OR "quitline" OR "text messaging" ) ) AND PUBYEAR > 2009 AND PUBYEAR < 2026 AND ( LIMIT-TO ( LANGUAGE , "English" ) ) AND ( LIMIT-TO ( EXACTKEYWORD , "Human" ) ) | | | 1,457 |
| Databases: Embase(Date of search: 11.13.2025) | | | | | | | | |
| Component | | | **Key word** | # | | **Search terms** | **Filters** | Results |
| Population | | | Tobacco product user  group terms | 1 | | ('combustible cigarette':ti,ab OR 'e-cigarette':ti,ab OR 'vaping':ti,ab OR 'dual use':ti,ab) |  | 14,868 |
| Intervention | | | Non-pharmacological cessation intervention  terms | 2 | | ('smoking cessation':ti,ab OR 'cognitive behavioral therapy':ti,ab OR 'motivational interviewing':ti,ab OR 'digital health':ti,ab OR 'quitline':ti,ab OR 'text messaging':ti,ab) |  | 97,407 |
| P & I | | | Population AND  Intervention | 3 | | #1 AND #2 |  | 1,740 |
| P & I + limits | | | Final (Identification count for PRISMA) | 4 | | #3 AND [english]/lim AND [humans]/lim AND [embase]/lim AND [2010-2025]/py | English; Humans; Embase records; Publication year  2010–2025 | 1,148 |
| Databases: Google Scholar(Date of search: 11.13.2025) | | | | | | | | |
| Component | | | **Key word** | # | | **Search terms** | **Filters** | Results |
| P & I (& optional SD) | | | All combined | 1 | | ("combustible cigarette" OR "e-cigarette" OR vaping OR "dual use")  AND  ("smoking cessation" OR "cognitive behavioral therapy" OR "motivational interviewing" OR "digital health" OR quitline OR "text messaging") | Custom range 2010–2025, English | 150 |

**Note:** Total records identified = 3,868 (databases: 3,718 [PubMed: 1,113 + Scopus: 1,457 + Embase: 1,148] plus other sources: 150 [Google Scholar]). In PubMed, publication date was limited at the day level (2010/01/01–2025/11/13[pdat]); Scopus and Embase were limited by publication year (2010–2025). For Google Scholar, the first 150 results sorted by relevance were screened, as this database does not support reproducible advanced filtering.

**Abbreviations:** CCU, combustible cigarette user; ECU, e-cigarette user; ti, title; ab, abstract; ti,ab, title/abstract (Embase); TITLE-ABS, title/abstract/keywords field (Scopus); MeSH, Medical Subject Headings.

# Table S2. Inclusion and Exclusion Criteria

| **Domain** | **Inclusion criteria** | **Exclusion criteria** |
| --- | --- | --- |
| Review scope | Studies relevant to conceptual synthesis of cessation-related research trends and clinical/public health implications | Studies not relevant to CCU/ECU/dual-use cessation-related synthesis |
| Population / user type | Targeted combustible cigarette users (CCUs), e-cigarette users (ECUs), or dual users | Studies not involving CCUs/ECUs/dual users as a primary user group |
| Intervention type | Behavioral and/or digital cessation interventions (e.g., cognitive behavioral therapy, motivational interviewing, digital health, quitline, text messaging), with or without adjunct pharmacotherapy. | Studies focused exclusively on pharmacological interventions without a behavioral/digital component. |
| Outcomes / study focus | Reported at least one of: user characteristics, intervention acceptability, or behavioral outcomes | Studies focused solely on secondhand smoke exposure or population-level policy effects without intervention-level evaluation |
| Language | English-language publications | Non-English publications |
| Publication years | 2010–2025 (database search); seminal works identified via hand-search regardless of publication year | Published outside 2010–2025 (unless identified as seminal via hand-search) |
| Information sources | PubMed, Scopus, Embase, and Google Scholar (search engine), plus Cochrane systematic reviews, U.S. Clinical Practice Guidelines for Treating Tobacco Use and Dependence, authoritative health organization reports | Sources outside defined databases and specified authoritative sources (if not relevant/eligible) |
| Study design / data availability | Empirical studies and evidence syntheses (systematic reviews/meta-analyses) reporting extractable data on user characteristics, intervention acceptability, and/or behavioral outcomes relevant to the narrative synthesis. | Protocols, commentaries, editorials, letters, or conference abstracts without original empirical data; studies with insufficient outcome data for synthesis. |

# Table S3. Characteristics and key findings of studies included in the narrative review and their thematic mapping

| **No** | **Author (year)** | **Study design** | **Population** | **User group tag** | **Intervention/exposure** | **Key findings** | **Mapped theme/section** |
| --- | --- | --- | --- | --- | --- | --- | --- |
| 1 | Abroms et al. (2014) | RCT | Adult CCUs (n=503), USA | CCU-only | Text2Quit (SMS program) vs self-help | 6-month abstinence: 11.1% vs 5.0% (RR 2.22, p<0.05). | 3.2.3; 3.3; 4.3.4 |
| 2 | Alshehri (2024) | Narrative review | CCUs attempting cessation | CCU-only | Traditional methods vs e-cigarettes | Mixed evidence for e-cigarettes as cessation tools due to safety concerns and lack of regulatory approval. | 1 |
| 3 | Bendotti et al. (2025) | Qualitative study | Quitline callers (CCUs/ECUs, n=30), Australia | Mixed/unclear | Motivational Interviewing (MI) | MI used in 94.7% of sessions with strong relational techniques but limited focus on ambivalence. | 3.2.2 |
| 4 | Benowitz (2010) | Narrative review | CCUs and nicotine-dependent users | CCU-only | Review of nicotine addiction mechanisms | Dependence is mediated by mesolimbic dopamine release, conditioning, and genetics. | 3.1.3; 4.2 |
| 5 | Blashki et al. (2008) | RCT | General practitioners (n=55), Australia | Out-of-scope | 20-hour CBT training program vs control | CBT training significantly improved practitioners' overall CBT competency scores (p=0.02). | 1 |
| 6 | Byaruhanga, Paul, et al. (2020) | RCT | Adult CCUs (n=430), rural Australia | CCU-only | Video vs telephone counselling | Video sessions were feasible but had higher odds of connectivity difficulties (OR 11.84). | 3.2.3 |
| 7 | Byaruhanga, Wiggers, et al.(2020) | RCT | Adult CCUs (n=655), rural Australia | CCU-only | Video vs telephone counselling vs self-help | Video was highly acceptable but had lower odds of reporting helpfulness vs telephone (OR 0.50). | 3.2.3 |
| 8 | Caponnetto et al. (2019) | Cohort study | CCUs (n=593), Italy | CCU-only | MI + pharmacotherapy | Varenicline plus MI showed the highest 52-week abstinence (46.8%). | 3.2.2; 3.3 |
| 9 | Carroll (2014) | Narrative review | Individuals with substance use disorders | Out-of-scope | Contingency management (CM) and CBT | Strong evidence supports CM/CBT, but routine clinical implementation remains limited. | 3.2.1; 4.3.1 |
| 10 | Carter et al. (2019) | Cohort study | CCUs with COPD/asthma (n=31,646), UK | Out-of-scope | Exposure: Respiratory disease status (observational) | Respiratory diseases in smokers were independently associated with cardiovascular diseases and mortality. | 1 |
| 11 | Cartujano-Barrera et al. (2020) | Quasi-experimental | Latino adult CCUs (n=50), USA | CCU-only | 12-week "Kick Butts" SMS program + NRT | At 12 weeks, 30% achieved biochemically verified abstinence with 90.9% satisfaction. | 3.2.3 |
| 12 | Çelik & Sevi (2020) | Systematic review/Meta-analysis | Adult CCUs | CCU-only | CBT with or without NRT | CBT is effective for smoking cessation, with efficacy further enhanced by pharmacotherapy. | 3.2.1; 3.3; 4.3.4; 4.4 |
| 13 | Chen et al. (2024) | RCT | Adult CCUs (n=206), China | CCU-only | CBT smartphone app vs minimal-contact | 4-week abstinence: 29.7% vs 6.7% (OR 5.92, p<0.001). | 3.2.4 |
| 14 | Colby et al. (2012) | RCT | Adolescent CCUs (n=162) | CCU-only | Enhanced MI vs brief advice | MI reduced cigarettes/day short-term but did not increase confirmed abstinence. | 3.2.2; 3.3; 4.3.1 |
| 15 | Cooper et al. (2016) | Cross-sectional study | Middle/high school students (n=3,704), USA | Mixed/unclear | Exposure: E-cigarette use (observational) | E-cigarette users had significantly higher odds of perceiving e-cigarettes as harmless. | 3.1.2; 3.3.2; 4.4 |
| 16 | DiClemente et al. (1991) | Cohort study | Adult CCUs (n=1,466), USA | CCU-only | Minimal self-help (stage-based) | Baseline stage of change strongly predicted quit attempts and abstinence at 6 months. | 3.2 |
| 17 | Erhabor et al. (2023) | Cross-sectional study | Adults (n=414,755), USA | Mixed/unclear | Exposure: E-cigarette use (observational) | Current e-cigarette use was 6.9%, with the highest prevalence among young adults aged 18–24. | 3.1.2 |
| 18 | Etter & Bullen (2011) | Cross-sectional study | ECUs (n=3,587) | Mixed/unclear | Exposure: E-cigarette use (observational) | Most users (92–96%) reported that e-cigarettes helped them quit or reduce smoking. | 3.1.2; 3.1.3 |
| 19 | Etter et al. (2023) | RCT | Daily CCUs (n=5,293), Switzerland/France | CCU-only | "Stop-Tabac" smartphone app | 6-month abstinence was predicted by dependence, motivation, app engagement/usefulness, and nicotine medication use. | 3.2.4 |
| 20 | Free et al. (2011) | RCT | CCUs aged ≥16 (n=5,800) | CCU-only | "txt2stop" SMS program vs control SMS | 6-month continuous abstinence: 10.7% vs 4.9% (RR 2.20, p<0.001). | 3.2.3; 3.3 |
| 21 | Gaiha et al. (2022) | Cross-sectional study | Adolescent/young adult ECUs (n=4,351), USA | ECU-only | Exposure: E-cigarette flavors (observational) | High youth use of disposable e-cigarettes and mint/menthol flavors indicates regulatory gaps. | 3.1.2 |
| 22 | Gill et al. (2022) | Cohort study | Male student CCUs aged 18-30 (n=127), India | CCU-only | MI sessions | 6-month cessation rate was 16.5%, predicted by high motivation and low nicotine dependence. | 3.2.2 |
| 23 | Goodchild et al. (2018) | Cross-sectional study | Adults ≥30 years across 152 countries | Out-of-scope | Exposure: Combustible smoking (observational) | Smoking costs 5.7% of global health expenditure, highlighting the economic need for cessation interventions. | 1 |
| 24 | Graham et al. (2021) | RCT | Young adult ECUs aged 18-24 (n=2,505), USA | ECU-only | "This is Quitting" (SMS program) vs assessment | 7-month 30-day abstinence: 24.1% vs 18.6% (OR 1.39, p<0.001). | 3.2.3; 4.3.3 |
| 25 | Graham et al. (2024) | RCT | Adolescent ECUs aged 13-17 (n=1,503), USA | ECU-only | "This is Quitting" (SMS program) vs assessment | 7-month 30-day abstinence: 37.8% vs 28.0% (RR 1.35, p<0.001) with no switch to combustible cigarettes. | 3.2.3; 4.3.2; 4.3.3 |
| 26 | Groom et al. (2021) | Cross-sectional study | Adolescent ever-ECUs aged 13-18 (n=1,549), USA | ECU-only | Exposure: Peer/media influence (observational) | Social media e-cigarette ads increased odds of friend-sourced initiation (aOR 2.04). | 3.1.2; 3.3.2; 4.4 |
| 27 | Harlow et al. (2022) | Cohort study | Adult dual users (n=1,544), USA | Dual | Exposure: Vaping motivation (observational) | Vaping motivation class did not predict 1-year cigarette abstinence (aOR 1.41). | 3.1.2; 3.1.3; 3.3.2 |
| 28 | Hartmann-Boyce et al. (2019a) | Systematic review/Meta-analysis | Adult smokers (predominantly CCUs; includes quitline/telephone counselling trials) | CCU-only | Telephone counselling (single/repeated; proactive/reactive) vs minimal support/usual care | Telephone counselling increased smoking cessation at longest follow-up (overall effect; stronger with proactive/repeated counselling; includes quitline settings | 3.2.3 |
| 29 | Hartmann-Boyce et al. (2019b) | Systematic review/Meta-analysis | CCUs using pharmacotherapy (83 RCTs) | CCU-only | Behavioral support vs less/no support | Behavioral support plus pharmacotherapy increased long-term abstinence (RR 1.15). | 3.3; 4.3.3; 4.3.4 |
| 30 | Hartmann-Boyce et al. (2021) | Systematic review/Meta-analysis | Adult CCUs (312 RCTs) | CCU-only | Behavioral interventions vs minimal intervention | Counseling (OR 1.44), financial incentives (OR 1.45), and SMS (OR 1.45) increased cessation. | 3.2; 3.3; 4.2; 4.4 |
| 31 | Heffner et al. (2025) | RCT | Young adult ECUs aged 18-30 (n=61), USA | ECU-only | "ACT on Vaping" (App+SMS) vs control SMS | 24-hour quit attempts: 87.5% vs 75.9%. Confirmed 30-day abstinence: 4.2% vs 0%. | 3.2.4 |
| 32 | Hettema et al. (2005) | Systematic review/Meta-analysis | Diverse CCUs and other populations (72 trials) | Mixed/unclear | Motivational Interviewing (MI) vs Controls | MI showed strong short-term effects (d=0.77) that decreased over time (d=0.30). | 3.2.2 |
| 33 | Imran Ho et al. (2025) | Systematic review/Meta-analysis | CCUs (15 RCTs) | CCU-only | Gamified interventions vs non-gamified controls | Gamified interventions improved short-term abstinence (RR 1.91, p<0.001). | 3.2.4 |
| 34 | Killen et al. (2008) | RCT | Adult CCUs ≥10 cigarettes/day (n=301) | CCU-only | Extended CBT vs brief telephone support | 20-week abstinence: 45% vs 29% (OR 1.98, p=0.006); 52-week difference was not significant. | 4.3.4 |
| 35 | Kim et al. (2019) | Cross-sectional study | Adult CCUs, ECUs, and dual users (n=2,333), Korea | Mixed/unclear | Exposure: E-cigarette use (observational) | Dual users reported higher willingness to quit (OR 1.35) with cessation as top motive. | 3.1.1; 3.1.2; 4.3.4 |
| 36 | Klemperer & Villanti (2021) | Cross-sectional study | Adult dual users with ≥1 quit attempt (n=204), USA | Dual | Exposure: Quit methods (observational) | Top motives to quit e-cigarettes were health (74%) and cost (45%). | 4.3.2 |
| 37 | Lancaster & Stead (2017) | Systematic review/Meta-analysis | CCUs (49 trials) | CCU-only | Face-to-face counseling vs minimal support | Individual counseling increased long-term cessation (RR 1.57). | 3.2.1; 3.3 |
| 38 | Lindson-Hawley et al. (2015) | Systematic review/Meta-analysis | CCUs (28 RCTs) | CCU-only | Motivational Interviewing (MI) vs brief advice | MI modestly increased ≥6-month cessation (RR 1.26), especially in primary care (RR 3.49). | 3.2.2; 3.3 |
| 39 | Lung et al. (2024) | Cross-sectional study | Adolescent gamers (n=2,362) | Mixed/unclear | E-cigarette education game | Game completion was 36.5%, showing high acceptability for e-cigarette prevention. | 3.2.4 |
| 40 | Marler et al. (2021) | Cohort study | Adult CCUs (n=212), USA | CCU-only | "Pivot" program (App + CO sensor + SMS) | 12-week continuous abstinence was 23.6%, and cigarettes/day decreased by 52.6%. | 3.2.4 |
| 41 | Marler et al. (2024) | Cohort study | Adult daily ECUs (n=75), USA | ECU-only | "Pivot" program (App + CO sensor + SMS) | 30-day abstinence was 45%, and dependence scores significantly decreased. | 3.2.4; 3.3.2; 4.3.1; 4.3.3; 4.3.4 |
| 42 | Martinez Agulleiro et al. (2023) | Narrative review | CCUs with serious mental illness (SMI) | CCU-only | Digital interventions vs standard care | Digital interventions show promise in SMI, achieving 19–24% abstinence rates. | 3.2.4 |
| 43 | Martinez et al. (2021) | RCT | Adult dual users (n=2,436), USA | Dual | Targeted self-help vs generic booklets vs assessment | Targeted self-help increased 3-month abstinence vs assessment only (d=0.20, p=0.012). | 4.3.2 |
| 44 | Martínez-Vispo et al. (2019) | RCT | Adult CCUs (n=204) | CCU-only | CBT + Behavioral Activation vs standard CBT | 12-month abstinence: 30% vs 24% (p>0.05). | 1 |
| 45 | Masaki et al. (2020) | RCT | Adult CCUs (n=584), Japan | CCU-only | CASC app + CO checker vs control app | Continuous abstinence (weeks 9–24): 62.3% vs 50.5% (OR 1.73, p=0.007). | 3.2.4 |
| 46 | Meltzer et al. (2021) | Qualitative study | Dual users (n=24) | Dual | Perceptions of self-help booklets | Identified specific vaping strategies and verified the appeal of adapted self-help materials. | 4.3.2 |
| 47 | Meza et al. (2023) | Cross-sectional study | Adults (n=363,568), USA | Out-of-scope | Exposure: Tobacco product use trends (observational) | Smoking prevalence decreased in young adults, but e-cigarette use increased significantly. | 3.1.1 |
| 48 | Morean et al. (2019) | Cross-sectional study | Adult ECUs (n=430) | ECU-only | Exposure: E-cigarette expectancies (observational) | Validated the 9-item Short E-Cigarette Expectancies Scale, correlating with vaping frequency. | 3.1.3; 4.2 |
| 49 | Palmer et al. (2022) | RCT | Young adult ECUs (n=27) | ECU-only | Telehealth Contingency Management (CM) vs monitoring | Confirmed abstinence: 80% vs 0% (p<0.001). | 3.3.2; 4.3.3; 4.3.4; 4.4 |
| 50 | Park et al. (2017) | Cross-sectional study | Adults (n=135,425), USA | Mixed/unclear | Exposure: Smoking to vaping switching (observational) | Exclusive e-cigarette use increased 100%, with switching higher among young adults. | 3.1.1; 3.1.3 |
| 51 | Perski et al. (2019) | RCT | CCUs using 'Smoke Free' app (n=57,214) | CCU-only | App with AI chatbot vs standard app | 1-month abstinence higher in chatbot group (OR 2.29, p<0.001) with higher engagement. | 3.2.4 |
| 52 | Pokhrel et al. (2015) | Qualitative study | Young adult daily ECUs (n=62) | ECU-only | Exposure: Motives for vaping (observational) | Identified 12 motives for vaping (e.g., cessation aid, health), suggesting motives beyond addiction. | 3.1.3; 4.2; 4.4 |
| 53 | Pokhrel et al. (2018) | Cross-sectional study | Young adult college students (n=410) | Mixed/unclear | Exposure: E-cigarette expectancies (observational) | Validated a short scale for e-cigarette expectancies; positive expectancies correlated with higher dependence. | 3.1.3 |
| 54 | Puljević et al. (2025) | Systematic review/Meta-analysis | Adult CCUs | CCU-only | SMS cessation interventions vs minimal intervention | SMS increased cessation (RR 1.37, 95% CI 1.25–1.50) with mixed results when adding pharmacotherapy. | 3.3 |
| 55 | Rajani et al. (2023) | Cohort study | Adult CCUs using 'Kwit' app (n=44) | CCU-only | Gamification features in 'Kwit' app | 7-day abstinence was 29%, with unlocking levels associated with higher abstinence odds (OR 1.22, p<0.001). | 3.2.4 |
| 56 | Rhoades et al. (2019) | Cross-sectional study | American Indian dual users (n=44), USA | Dual | Exposure: Dual use patterns (observational) | Higher dependence on combustible cigarettes than e-cigarettes, with 71% using zero/low-nicotine e-liquids. | 3.1.2; 4.3.4 |
| 57 | Roth et al. (2020) | Cross-sectional study | Global population (204 countries) | Out-of-scope | Exposure: Global tobacco use (observational) | Tobacco remains a leading global risk factor, emphasizing the critical need for cessation interventions. | 1 |
| 58 | Rüther et al. (2018) | RCT | Outpatient CCUs (n=100) | CCU-only | "Smoke_less" CBT program vs active control | Abstinence higher at 1 week (OR 7.00) and 5 months (OR 7.31) with no significant effect at 12 months. | 3.2.1 |
| 59 | Sanchez et al. (2021) | Qualitative study | Youth/young adult ECUs wanting to quit (n=41) | ECU-only | Exposure: Vaping cessation perceptions (observational) | Identified unique quitting barriers: high nicotine dependence, ubiquity of devices, and lack of self-awareness. | 3.2.1; 3.3.2; 4.3.1; 4.3.3; 4.3.4; 4.4 |
| 60 | Shi et al. (2022) | Cross-sectional study | Middle/high school students (n=8,471), Canada | Mixed/unclear | Exposure: Vaping predictors (observational) | Machine learning predicted vaping with high accuracy (AUC 0.83) based on age, peer vaping, and alcohol use. | 3.2.4 |
| 61 | Sikka et al. (2021) | Case series | Daily ECUs (n=6) | ECU-only | Counseling + pharmacotherapy (NRT/bupropion) | 6-month cessation achieved by 3/6 patients, with barriers including stress and peer influence. | 3.3.2 |
| 62 | Skov-Ettrup et al. (2016) | RCT | Daily CCUs (n=1,813), Denmark | CCU-only | Proactive vs reactive telephone counselling | 12-month prolonged abstinence: 11.0% vs 6.9% (OR 1.62, p=0.004). | 3.2.3 |
| 63 | Skov-Ettrup et al. (2014) | RCT | Daily CCUs (n=8,624), Denmark | CCU-only | Internet vs proactive/reactive phone vs booklet | Proactive phone counselling had highest uptake (74%) and was favored by those with lower education. | 3.3 |
| 64 | Stead et al. (2017) | Systematic review/Meta-analysis | Adult CCUs (66 trials) | CCU-only | Group behavior therapy vs self-help/no intervention | Group therapy more effective than self-help (RR 1.88) and no intervention (RR 2.60). | 3.2.1; 4.3.1; 4.3.4 |
| 65 | Stead et al. (2012) | Systematic review/Meta-analysis | Adult CCUs (n=19,488; 53 trials) | CCU-only | Pharmacotherapy + behavioral support vs usual care | Combined treatment increased cessation (RR 1.83, 95% CI 1.68–1.98). | 4.3.1 |
| 66 | Thomson & Islami (2024) | Cohort study | Adults (n=430,015), USA | Out-of-scope | Exposure: Quitting smoking vs current smoking (observational) | Quitting avoided substantial excess mortality within 1–9 years: 63% (cardiovascular), 53% (cancer). | 1 |
| 67 | Tindle et al. (2018) | Cohort study | Participants (n=8,907), USA | Out-of-scope | Exposure: Years since quitting (observational) | Lung cancer risk dropped 39.8% within 5 years of quitting but remained higher than never smokers. | 1 |
| 68 | Tzelepis et al. (2019) | Systematic review/Meta-analysis | Adult CCUs (n=615; 2 RCTs) | CCU-only | Video counselling vs telephone counselling | No significant difference in cessation (RR 1.15, p=0.38). | 4.3.3; 4.3.4 |
| 69 | Vickerman et al. (2021) | RCT | Dual users calling a quitline (n=96) | Dual | Enhanced E-cigarette Coaching (EEC) vs TAU | Most EEC participants chose quit plans combining NRT and vaping.;4.2.2 | 4.3.2 |
| 70 | Vinci (2020) | Narrative review | CCUs (special populations) | CCU-only | CBT and Mindfulness-based interventions (MBIs) | CBT and MBIs show initial efficacy via telehealth and combined with pharmacotherapy for diverse populations. | 3.2; 3.2.1; 3.3; 4.3.1 |
| 71 | Wang et al. (2023) | Cross-sectional study | Adults (n=30,736), USA | Mixed/unclear | Exposure: E-cigarette use (observational) | Exclusive e-cigarette users had higher odds of moderate (aOR 1.63) and severe (aOR 2.18) psychological distress. | 3.1.3 |
| 72 | Webb et al. (2023) | Quasi-experimental | Adult ECUs (n=51) | ECU-only | 6-week mHealth program (NRT + CBT) | 1-month 30-day abstinence: 29.4%; continuous 30-day abstinence: 25.5%. | 3.2; 3.3.2; 4.3.1; 4.3.3; 4.3.4; 4.4 |
| 73 | Webb et al. (2022) | RCT | Adult CCUs (n=259) | CCU-only | Digital clinic (App CBT + coaching + NRT) vs brief advice | 52-week CO-verified abstinence: 22.9% vs 10.2% (RR 2.25). | 3.2.4 |
| 74 | Webb et al. (2020) | RCT | Adult CCUs (n=546) | CCU-only | Digital clinic (App CBT + coaching + NRT) vs brief advice | 4-week 7-day point prevalence abstinence: 44.5% vs 28.1% (RR 1.58, p<0.001). | 3.2.4 |
| 75 | Weser et al. (2021) | Quasi-experimental | Adolescents (n=345), USA | Mixed/unclear | VR prevention game vs TAU | VR game significantly improved e-cigarette knowledge and harm perceptions vs control. | 3.2.4 |
| 76 | Whittaker et al. (2019) | Systematic review/Meta-analysis | CCUs (n=33,849; 26 RCTs) | CCU-only | Mobile phone interventions vs minimal support | Automated SMS increased cessation vs minimal support (RR 1.54); smartphone apps showed no clear benefit (RR 1.00). | 3.2; 3.2.3; 3.3; 4.3.1; 4.3.3; 4.3.4; 4.4 |
